# New insights into the molecular phylogeny, biogeographical history, and diversification of *Amblyomma* ticks (Acari: Ixodidae) based on mitogenomes and nuclear sequences

**DOI:** 10.1186/s13071-024-06131-w

**Published:** 2024-03-18

**Authors:** Juan E. Uribe, Samuel Kelava, Santiago Nava, Andrea P. Cotes-Perdomo, Lyda R. Castro, Fredy A. Rivera-Paéz, Silvia Perea, Ben J. Mans, Alexander Gofton, Ernest J. M. Teo, Rafael Zardoya, Stephen C. Barker

**Affiliations:** 1 Biodiversity and Evolutionary Biology Department (BEBD), Museo Nacional de Ciencias Naturales (MNCN-CSIC), Madrid, Spain; 2 Invertebrate Zoology Department, National Museum of Natural History, Smithsonian Institution, Washington, DC USA; 3 Department of Parasitology, School of Chemistry and Molecular Biosciences, The University of Queensland, Brisbane, QLD Australia; 4 Estación Experimental Agropecuaria Rafaela (EEA Rafaela), Instituto Nacional de Tecnología Agropecuaria, Santa Fe, Argentina; 5 Department of Natural Sciences and Environmental Health, Faculty of Natural Sciences and Maritime Sciences of Technology, University of South-Eastern, Bø i Telemark, Norway; 6 Grupo de Investigación Evolución, Sistemática y Ecología Molecular (GIESEMOL), Facultad de Ciencias Básicas, Universidad del Magdalena, Santa Marta, Colombia; 7 Grupo de Investigación en Genética, Biodiversidad y Manejo de Ecosistemas (GEBIOME), Departamento de Ciencias Biológicas, Facultad de Ciencias Exactas y Naturales, Universidad de Caldas, Calle 65 No. 26-10, 170004 Manizales, Caldas Colombia; 8 Epidemiology, Parasites and Vectors, Agricultural Research Council–Onderstepoort Veterinary Research, Onderstepoort, South Africa; 9 Department of Life and Consumer Sciences, University of South Africa, Pretoria, South Africa; 10 CSIRO Health and Biosecurity, Canberra, ACT Australia

**Keywords:** Ixodidae, Metastriata, Hard ticks, Pathogen vectors, Mitogenomics, Time-tree

## Abstract

**Background:**

*Amblyomma* is the third most diversified genus of Ixodidae that is distributed across the Indomalayan, Afrotropical, Australasian (IAA), Nearctic and Neotropical biogeographic ecoregions, reaching in the Neotropic its highest diversity. There have been hints in previously published phylogenetic trees from mitochondrial genome, nuclear rRNA, from combinations of both and morphology that the Australasian *Amblyomma* or the Australasian *Amblyomma* plus the *Amblyomma* species from the southern cone of South America, might be sister-group to the *Amblyomma* of the rest of the world. However, a stable phylogenetic framework of *Amblyomma* for a better understanding of the biogeographic patterns underpinning its diversification is lacking.

**Methods:**

We used genomic techniques to sequence complete and nearly complete mitochondrial genomes –ca. 15 kbp– as well as the nuclear ribosomal cluster –ca. 8 kbp– for 17 *Amblyomma* ticks in order to study the phylogeny and biogeographic pattern of the genus *Amblyomma*, with particular emphasis on the Neotropical region. The new genomic information generated here together with genomic information available on 43 ticks (22 other *Amblyomma* species and 21 other hard ticks–as outgroup–) were used to perform probabilistic methods of phylogenetic and biogeographic inferences and time-tree estimation using biogeographic dates.

**Results:**

In the present paper, we present the strongest evidence yet that Australasian *Amblyomma* may indeed be the sister-group to the *Amblyomma* of the rest of the world (species that occur mainly in the Neotropical and Afrotropical zoogeographic regions). Our results showed that all *Amblyomma* subgenera (*Cernyomma*, *Anastosiella*, *Xiphiastor*, *Adenopleura*, *Aponomma* and *Dermiomma*) are not monophyletic, except for *Walkeriana* and *Amblyomma*. Likewise, our best biogeographic scenario supports the origin of *Amblyomma* and its posterior diversification in the southern hemisphere at 47.8 and 36.8 Mya, respectively. This diversification could be associated with the end of the connection of Australasia and Neotropical ecoregions by the Antarctic land bridge. Also, the biogeographic analyses let us see the colonization patterns of some neotropical *Amblyomma* species to the Nearctic.

**Conclusions:**

We found strong evidence that the main theater of diversification of *Amblyomma* was the southern hemisphere, potentially driven by the Antarctic Bridge's intermittent connection in the late Eocene. In addition, the subgeneric classification of *Amblyomma* lacks evolutionary support. Future studies using denser taxonomic sampling may lead to new findings on the phylogenetic relationships and biogeographic history of *Amblyomma* genus.

**Graphical Abstract:**

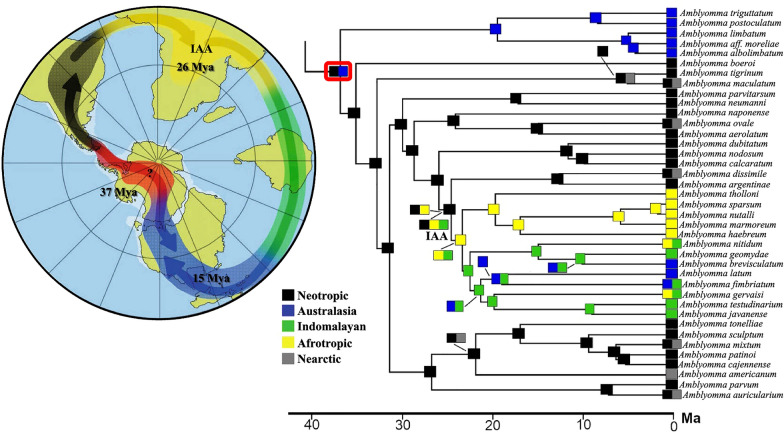

**Supplementary Information:**

The online version contains supplementary material available at 10.1186/s13071-024-06131-w.

## Background

Ticks (Arachnida: Ixodida) are highly specialized, blood-sucking ectoparasites of vertebrates [1]. They are one of the main pathogen vectors (bacteria, viruses and protozoa) in the world, causing a expansive range of infections and diseases (such as rickettsioses, Lyme disease, relapsing fever borrelioses, Crimean-Congo hemorrhagic fever, babesioses, among others), and are therefore of maximal economic and health importance [2–5]. The order Ixodida comprises approximately 970 living species that are classified into three families, Nuttalliellidae (monotypic), Argasidae, and Ixodidae [6, 7]. Ixodidae (hard ticks) is the most diversified one with 771 valid species that are classified into two groups, Prostriata (with a single genus, *Ixodes*) and Metastriata (including 14 extant genera plus two additional fossil genera, *Compluriscutula* and *Cornupalpatum* [6]). Within Metastriata, *Amblyomma* is the second most diversified genus (after *Haemaphysalis*), comprising 136 living species, which are widely distributed across the Indomalayan, Afrotropical, Australasian, Nearctic and Neotropical biogeographic regions, reaching its highest diversity in the Neotropics region (67 species) [8]. The wide distribution, high diversity, and economic and health importance make the genus *Amblyomma* a priority group for evolutionary and biogeographical studies.

For many years, the genera *Amblyomma* and *Aponomma* were grouped together in the subfamily Amblyomminae or in the subtribe Amblyommini [9, 10]. However, Dobson and Barker [11], using DNA sequencing, determined that the genus *Aponomma* was paraphyletic and, consequently, also the subfamily Amblyomminae. “Indigenous” and “primitive” *Aponomma* species sensu Kaufman [12] were recovered as independent lineages and assigned to their own genera, *Bothriocroton* [13], *Robertsicus* and *Archaeocroton* [14], while “typical” *Aponomma* species sensu Kaufman [12] were transferred to the *Amblyomma* genus [13]. Recently, *Amblyomma transversale* has emerged as an early and independent branch in the mitogenomic tree of Metastriata, and has been elevated to the genus level, namely *Africaniella* [15]. As a consequence of the numerous taxonomic changes in the group, the current *Amblyomma* diversity seems to retain plesiomorphic characters that make the taxonomic boundaries diffuse. This diffuse state also hinders the assignment of fossils to a given cladogenetic event for estimation of their ages of diversification. Likewise, the most robust evolutionary framework for these lineages (based on mitochrondrial [mt] genomes) is not complete enough to either establish potential diversification events or to explain the current wide tropical distribution of the Metastriata group [16].

Various studies have been conducted with the aim to understand the evolutionary patterns of the *Amblyomma* species and the potential historical events that have driven their diversification [16–28]. However, a robust phylogeny of the genus is still lacking. Additionally, the subgeneric classification of *Amblyomma* sensu Santos Dias [29] and Camicas et al. [30] (9 subgenera: *Adenopleura*, *Amblyomma*, *Anastosiella*, *Aponomma*, *Cernyomma*, *Dermiomma*, *Haemalastor*, *Walkeriana* and *Xiphiastor*) has shown to be particularly weak from a phylogenetic perspective [16–18, 31].

Hence, evolutionary studies of *Amblyomma* remain inconclusive although weakly supported available phylogenetic frameworks of the genus show that early divergences are restricted to species endemic of Australian and the southern Neotropical regions [23, 24, 26]. Furthermore, IAA (Indomalayan, Afrotropical and Australasian biogeographic ecoregion) species are recovered in a derived phylogenetic position, together with a wide polytomy across main American lineages [26]. Thus far, genomic sequence data and taxon sampling are sparse, precluding the existence of a robust biogeographical hypothesis for hard ticks, especially in the Neotropic region, where the highest diversity of *Amblyomma* (67 species) is found.

At present, 316 mt genomes are available for the order Ixodida (at August 2023; [16, 31, 32]), but these only cover 20 species of *Amblyomma*, far from representing the diversity of the genus, or at least its main lineages. Therefore, the aim of this study was to generate a balanced mitogenomic dataset to reconstruct, using probabilistic methods, a robust and stable phylogenetic framework of *Amblyomma* for a better understanding of the evolutionary history and the biogeographic patterns underpinning its diversification, with particular emphasis on the Neotropical region.

## Methods

### Sample collection, DNA extraction and sequencing

A total of 17 *Amblyomma* species were collected and processed in this study (see Additional file 1: Table S1 for a complete list, localities, collector and sampling date of each of these species). All samples were fixed in absolute ethanol and stored at - 20ºC. After morphological identification, total DNA was extracted using the Qiagen DNeasy Blood & Tissue Kit (Qiagen, Hilden, Germany) following the manufacturer’s spin-column protocol.

Using universal primers [33], we amplified the partial fragment of mt cytochrome *c* oxidase I (COXI) by PCR and performed Sanger sequencing with the aim of molecular re-confirmation. The mt genomes of five species were subjected to long-range PCR amplification using specific primers based on the partial COX1 fragment and conserved regions using the reaction contents and thermocycler conditions described by Cotes-Perdomo et al. [28]. The long-range PCR products were purified by ethanol precipitation, and cleaned fragments from the same mt genome were pooled together in equimolar concentrations, followed by shearing to an average fragment size of 450 bp using the Covaris ME220 Focused-ultrasonicator (Covaris, Woburn, MA, USA). The library preparation and massive parallel sequencing protocol were performed at the Laboratories of Analytical Biology (LAB-NMNH) as described in Uribe et al. [25]. For each remaining sample, a TruSeq Nano DNA Library was constructed and subjected to whole-genome sequencing (WGS) on the NovaSeq 6000 platform (150PE, 18 Gb/ea; Macrogen®, Seoul, Korea). The sequencing methods of the samples are described in Additional file 1: Table S1, and the primers and amplification strategy are described in Additional file 2: Table S2.

### Data curation and ortholog search

Three types of massive parallel sequencing were combined in this study, namely amplicons, WGS and RNA sequencing (RNA-Seq) (available in NCBI; see Additional file 1: Table S1), for the configuration of the matrices. The quality of the massive sequencing was assessed using FastQC version 0.10.1 [34]. All raw reads were trimmed and cropped, and adapters were removed using Trimmomatic version 0.39 [35]. Paired-end cleaned reads were *de novo* assembled with SPAdes version 3.14.0, using spades.py [36] and rnaspades.py [37]. For the amplicon sequencing and WGS, the assembled mt genomes for each species were filtered by BLAST version 2.6.0 [38] searching against a custom database created using all *Amblyomma* mt genomes available. Then, the previously sequenced COX1 of each sample was used to corroborate the identifications. All individualized mt genomes were reference mapped using the paired-end cleaned reads with Geneious® in order to visually check the assemblies and determine the mean depth coverage. Mitochondrial elements were annotated using MITOS [39] and the open reading frames (ORFs) were manually checked. The boundaries of ribosomal genes were determined following Boore et al. [40].

### Construction of matrices and phylogenetic inferences

A total of 60 mt genomes (including 17 new mt genomes of *Amblyomma*) were used to construct matrices, where three *Ixodes* species were used as outgroup of Metastriata. The complete list of matrices and their features are shown in Additional file 3: Table S3. The protein-coding genes were aligned using nucleotide sequences guided by the deduced amino acids in the Translator X server [41] with MAFFT v5 [42]. The nucleotide sequences of both ribosomal RNA (rRNA) genes were aligned using MAFFT version 7 [43] with default parameters. Additionally, a nuclear matrix was contracted using the ribosomal cluster for 12 *Amblyomma* species that represent all biogeographic regions.

Phylogenetic relationships were inferred using maximum likelihood (ML; [44]) and Bayesian inference (BI; [45, 46]). BI analyses were performed using two software programs: (i) under MrBayes v.3.1.2. [47], four simultaneous Markov chain Monte Carlo (MCMC) chains for 20 million generations were run, with sampling every 1000 simulated samples and discarding the first 25% as burn-in to prevent sampling before reaching stationarity; and (ii) under PhyloBayes [48], the site-heterogeneous model of evolution CAT-GTR model was selected [49], and two independent MCMC chains were run, with sampling every cycle, until convergence (checked a posteriori using the tools implemented in PhyloBayes; maximum difference < 0.1, maximum discrepancy < 0.1, effective sample size > 100). Consensus trees and the posterior probabilities (PP) of the nodes were obtained after discarding the first 10% cycles. The convergence of MCMC chains was checked and determined in Tracer v.1.7 [50]. ML analyses were carried out in IQ-TREE v.1.6.1 using a combination of rapid hill-climbing and stochastic perturbation methods. A total of 1000 ultrafast bootstrap pseudoreplicates were performed to assess robustness of the inferred trees, including the percentage of bootstrap (PB) of the nodes.

Two matrices were constructed, one at the amino acid codification and one at the nucleotide codification. For the concatenated amino acids matrix, the best-fit amino acid replacement matrices (Rmatrix) were selected under Bayesian Information Criterion (BIC; [51]) using the command ‘–m TEST’ in ModelFinder [52]; the fit of adding empirical frequency profiles (mixture model) to the selected amino acid replacement matrices was then tested using the BIC and the command ‘–mset Rmatrix+C10, Rmatrix+C20, Rmatrix+C30, Rmatrix+C40, Rmatrix+C50, Rmatrix+C60’ in ModelFinder. The nucleotide matrix was analyzed, concatenated and partitioned (by codon position + 12S and 16S rRNA). The best evolutionary model for the concatenated matrix was selected with ModelFinder under BIC, and the ‘-m TESTNEW’ command, while for the partitioned matrices, the command line was ‘-m TESTMERGEONLY’ (which also infers the best scheme partition). All models and the scheme selected are described in Additional file 3: Table S3.

### Time-tree estimation

The BEAST 2.6.6 program [53] was used to perform a Bayesian estimation of divergence times among *Amblyomma* species, based on the mt genome using protein coding + ribosomal genes at nucleotide codification (subset of Matrix BI-NT-GBlock-Partitioned; see Additional file 3: Table S3). An uncorrelated relaxed molecular clock was used to infer branch lengths and nodal ages. The tree topology was set based on topology 2 (see Additional file 5: Fig. S1b) using *Dermacentor*, *Hyalomma*, *Rhipicephalus* and *Rhipicentor* as outgroups. For the clock model, the lognormal relaxed-clock model was selected, which allows rates to vary among branches without any a priori assumption of autocorrelation between adjacent branches. For the tree prior, a Yule process of speciation was employed. ModelFinder was used as indicated above to select the best-fit partition and evolutionary models (Additional file 3: Table S3). The final Markov chain was run twice for 100 million generations, with sampling every 10,000 generations, and the first 10 million generations were discarded as part of the burn-in process, according to the convergence of chains checked with Tracer v. 1.7.1. The effective sample size of all the parameters was > 200.

For the time estimations, we used a phylogenetic scenario previously reconstructed (also inferred here) as a scaffold to include dates of biogeographic events [20, 22, 28]. The posterior distribution of the estimated divergence times was obtained by specifying four calibration points as priors, which are highlighted as follows.

1. A first calibration point was set for the divergence of *Am. americanum* + *Am. cajennense* group, at between 25 and 15.2 million years ago (Mya), with a mean of 20 Mya (normal distribution; 3 Sigma, 0.01 Offset; 95% height posterior density [HPD]) [20].
2. A second calibration point was set to *Am. cajennense* group radiation with a mean of 17 Mya (normal distribution; 0.3 Sigma, 0.001 Offset; 95% HPD) [22].
3. A third calibration point was set for the *Am. mixtum* (*Am. cajennense* + *Am. patinoi*) cladogenetic event, with a mean of 6 Mya (normal distribution; 0.6 Sigma, 0.001 Offset; 95% HPD between 5.01 and 6.99 Mya) [20].
4. A fourth calibration point was set for the divergences of the *Am. maculatum* + *Am. tigrinum* cladogenetic event at between 3.3 and 0.899 Mya, with a mean of 2.1 Mya (normal distribution; 0.73 Sigma; 95% HPD) [22].

### Biogeographical analyses

The biogeographical history of the genus *Amblyomma* was reconstructed using BioGeoBEARS [54] implemented in R. Four biogeographical models implemented in BioGeoBears were evaluated: DEC (Dispersal-Extinction-Cladogenesis [55]) and DIVA+like, plus these two models considering the “j” parameter (founder-event speciation), DEC+J and DIVALIKE+J [54]. Accounting for founder-event speciation is relevant when considering organisms such as *Amblyomma* due to their great relevance as vectors and their potential distribution changes by climate change and anthropogenic pressure (but see Ree and Sanmartin [56] for j parameter criticism). Biogeographic models were compared using the corrected Akaike information criterion (AICc) to determine the best-fit biogeographic model on data and the effect of adding the “j” parameter on model performance. For biogeographic analyses, the distribution range was divided in five areas that were defined based on the distribution of the species included (Nearctic [NE], Neotropic [NO], Australia [AU], Afrotropic [AF] and Indomalayan [IN]). Given that the evolutionary relationships of Metastriate are not resolved, no area was assigned to the outgroup (see Additional file 4: Table S4). The broad geographic scope of this study justifies the consideration of such large areas for computational efficiency and to achieve the reconstruction of the biogeographical history of the whole genus *Amblyomma*.

## Results and Discussion

Ticks are the second most important vectors of disease globally and potentially one of the most critical organisms to be studied in every biological field. Establishing a solid evolutionary framework of *Amblyomma* within the Metastriata context may be key to understanding the origin of the diversification of the genus. In this study, we used the leafier tree (in number of species) published recently [26] to select the species representatives of each main lineage. Thus, the molecular dataset generated here is described and, for the first time at the mitogenomic level, used to reconstruct *Amblyomma* phylogenetic relationships to assess previous hypotheses related to the origin and patterns of diversification of the genus.

### Mitogenomic features

A total of 17 mt genomes (3 of which were partial) were newly sequenced in this study, increasing the mt genome catalog available for this tick group to 39 genomes (from 22). The sequencing methods applied here to determine the mt genomes (amplicon and WGS) allowed us to obtain a sequencing depth similar to that reached in related articles recently published [16, 28] (see depth coverage in Additional file 1: Table S1). The features of the new complete mt genomes described here fit with those already reported for other mt genomes of Metastriata [57], including the gene order (except for *Africaniella tranversale* [27]), length of the genomes/genes and the two characteristic control regions. Also, a general feature of the 37 genes (13 protein-coding genes, 22 tRNAs and two rRNAs) fit with most metazoan mt genomes [40].

### Phylogeny of Amblyomma

**Fig. 1:**
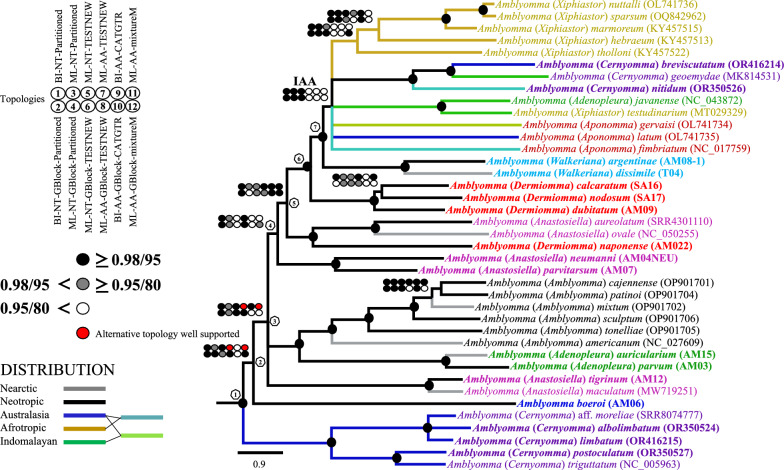
Phylogenetic hypotheses for *Amblyomma* relationships based on the mitogenomic datasets. Two matrices were assessed using the coding protein genes at amino acid codification (AA) and at nucleotide codification (NT; this last includes both ribosomal genes). Trees were reconstructed using maximum-likelihood (ML) and Bayesian inference (BI) applying 6 treatments to each matrix (these are described in the left top of the figure and in section Methods section). Circles (left of tree) refer to the credibility support of the nodes, with the black-gilled circle indicating strong support; the gray-filled circle, moderate support; and the open circle, no significant support (at the left middle of figure). All trees were summarized based on the following criteria. If in at least half of the trees a given node is not recovered with significant statistical support (node ≥ 0.95 PP/80 PB), it is collapsed (see all topologies in Additional file 1: Fig. S1a–l). Black-filled circles at a given node indicate nodes with maximum bootstrap and PB values across the 12 inferences. The geographical distribution of the species included in the tree are indicated with color coding in their respective branches, as is described in the lets button. The scale bar is given in expected substitutions/site. The specimens sequenced in this study are highlighted in bold font, and species with the same subgeneric status are indicated in the same color. The lineage with Indomalayan, Afrotropical and Australasian distributions is indicated in the tree with IAA

To reconstruct the internal relationships of *Amblyomma*, we inferred 12 phylogenetic trees in this study, six at the nucleotide level and six at the amino acid level. All trees were summarized in Fig. 1, where the nodes that were not recovered in at least half of the trees with significant statistical support (node ≥ 0.95 PP/80 PB) were collapsed (see all topologies in Additional file 5: Fig. S1a–l). Also, dataset features, best-fit partition schemes and models, and log-likelihood values of all phylogenetic analyses are provided in Additional file 3: Table S3. *Amblyomma* was recovered as monophyletic in all analyses (Fig. 1, node 1) and as sister to a clade that includes (*Dermacentor* ((*Rhipicentor* (*Hyalomma* + *Rhipicephalus*))), as has been widely reported in previous phylogenies [27, 57].

All topologies but two (i.e. Topology 7 and 11) are congruent in the early cladogenetic events of *Amblyomma*. Topology 7 and 11 (see Additional file 5: Fig. S1g, k) recovered the *Am. maculatum* complex as a sister clade of *Amblyomma boeroi* with significant and moderate support. The rest of the trees recovered endemic Australian species as a sister clade of *Am. boeroi* (southern cone of South America) + the rest of *Amblyomma* lineages. No comparable results have been reached so far in the phylogenetic analyses (Fig. 1, node 2). Although Seabolt [23], Beati and Klompen [24], and Santodomingo et al. [26] recovered three endemic *Amblyomma* species from the southern cone of South America (*Am. boeroi*, *Am. parvitarsum* and *Am. neumanni*) and Australian endemic species as the first divergences of *Amblyomma*, these relationships differ between them. Beati and Klompen [24] recovered a sister-relationship between *Am. boeroi* and *Am. parvitarsum*, while Santodomigo et al. [26] and Seabolt [23] recovered *Am. neumanni* + *Am. parvitarsum* as a sister lineage of *Am. boeroi*, an endemic Australian clade in an early phylogenetic position, but without statistical support. In our analyses, *Am. neumanni* and *Am. parvitarsum* are recovered as sister species but in a derivative position with strong support (Fig. 1, node 4). The close phylogenetic relationship between *Am. parvitarsum* and *Am. neumanni* was previously reported [58]. *Amblyomma boeroi* was recovered as an independent phylogenetic lineage within the genus *Amblyomma*, but it shares some morphological characters with both *Am. parvitarsum* and *Am. neumanni* [19]. On the other hand, some endemic species of Australia were recovered as the first divergence, as sister clade of all other species of *Amblyomma* in all phylogenetic trees (Fig. 1). Our results are compatible with those from previous *Amblyomma* phylogenetic analyses (although they differ in some included endemic species of Australia) that recovered *Am. fimbriatum* and *Am. postoculatum* as the sister clade that includes *Am. moreliae*, *Am. albolimbatum* and *Am. limbatum* [24]. The species clustered in this Australian clade are mostly associated with reptile hosts, with the exception of *Am. postoculatum*, which parasitize the wallaby banded hare [59].

In node 3 (Fig. 1), a tripartite polytomy exists between the* Am. maculatum* group, which does not appear to be closely related to any other species included in our analyses (except in Topology 7 and 11, see above). Also in node 3, (*Am. parvum* + *Am. auricularium*) + (*Am. cajennense* group + *Am. americanum*) relationships are well supported. These relationships have been recovered in previous studies (clades G, H, I in [26]). The internal relationships within the *Am. cajennense* complex as well as its sister relationships with *Am. americanum* are congruent with previous phylogenetic analyses [20, 26, 28]; however, the related species to *Am. americanum* should be included in a mitogenomic framework to be discussed in future studies. Lastly, node 3 includes (*Am. neumanni* + *Am. parvitarsum*) + the remaining species, including Neotropical, Neotropical-Nearctic (as *Am*. *dissimile*) and IAA species.

In the clade that includes the remaining species of *Amblyomma*, successive relationships with strong support were recovered between nodes 5, 6 and 7 (Fig. 1), as follows:

- ((*Amblyomma aureolatum*, *Am. ovale*), *Am. naponense*) (Fig. 1). The close relationship between *Am. aureolatum* and *Am. ovale* was identified in previous studies [17, 25], as was that with *Am. naponense* (clade E [26, 60]). *Amblyomma aureolatum* and *Am. ovale* normally use members of order Carnivora as hosts for adult stages (despite both species having a more widespread range of hosts) and are morphologically similar. Both factors have led to confusion regarding their identification. *Amblyomma ovale* is more widely distributed (Nearctic and Neotropic) than *Am. aureolatum* (Neotropic) [8].
- ((*Amblyomma calcaratum*, *Am. nodosum*), *Am. dubitatum*) (Fig. 1). These relationships were previously recovered [26, 61, 62], but in our analyses the relationship of *Am. calcaratum* and *Am. nodosum* was not recovered in four of 12 trees. This is likely due to insufficient taxon sampling of the species related to this clade in our analyses, as *Am. yucumense* (normally associated to *Am. dubitatum*), *Am. hadanii* and *Am. coelebs* [26, 62]. Note that adults of *Am. nodosum* and *Am. calcaratum* have been often confused because they are normally associated with hosts in the Pilosa order (e.g*. Myrmecophaga tridactyla*, *Tamandua tetradactyla, T. mexicana*), although both have other hosts throughout their life-cycles [8].
- ((*Am*. *calcaratum*, *Am*. *nodosum*), *Am*. *dubitatum*) was recovered as sister linage of ((*Am*. *argentinae* + *Am*. *dissimile*) + the IAA species) with confident support in all topologies (node 6 and 7; Fig. 1). However, (*Am*. *argentinae* + *Am*. *dissimile*) as a sister clade of the IAA species was only recovered in the analyses with nucleotide codification (6 of out 12; Fig. 1). In the amino acid matrix, (*Am*. *argentinae* + *Am*. *dissimile*) appears in a wide polytomy with IAA species (see inset, Fig. 1). *Amblyomma argentinae* and *Am. dissimile* were recovered closely related to IAA species in a previous study but without statistical support [26]. The lack of taxonomic sampling may be the cause of the loss of the phylogenetic signal in this part of the phylogenetic assembly.

With regard to IAA species, three well-supported clades were recovered in our analyses: (i) (*Am*. *javanense* + *Am. testudinarium*), as was also recovered in Uribe et al. [25]; (ii) the ((*Am*. *breviscutatum* + *Am. geoemydae*) *Am. nitidum*) clade, recovered for the first time with strong support in all topologies; *Am. geoemydae* and *Am. nitidum* have a Indomalayan distribution associated mostly to reptiles [63, 64], while *Am*. *breviscutatum* occurs in Australia and the southwestern Pacific on feral pigs and rats (as nymphs) [59, 65]; and (iii) the ((((*Am. nuttallli* + *Am. sparsum*) *Am. marmoreum*) *Am. hebraeum*) *Am. tholloni*) clade, which is well supported in seven out of 12 topologies (Fig. 1). This clade has also been reported in mitogenomic analyses [27, 66], it has an Afrotropical distribution and includes ticks with public health importance, as are the species of the marmoreum complex [66]. Finally, the increasing taxon sampling of the “typical” *Aponomma* forms (*Am. gervaisi, Am. latum* and *Am. fimbriatum*) supports the inclusion of these species in *Amblyomma* (see [13]) with strong statistical support, even though that their phylogenetic positions remain unresolved (Fig. 1).

The results of the present study are in agreement with those from previous analyses [16–18, 67], showing that most of the *Amblyomma* subgenera sensu Camicas et al. [67] and Santos Dias [29] are not monophyletic, as in the cases of *Cernyomma, Anastosiella, Xiphiastor, Adenopleura, Aponomma,* and *Dermiomma. Amblyomma* (as in [26, 28]) and *Walkeriana* were found to be the only monophyletic subgenera.

Finally, the nuclear tree (Additional file 6: Fig. S2) recovered a monophyletic *Amblyomma* but with a polytomy in its early relationships that involves *Am. boeroi*, *Am. postoculatum*, *Am. neumanni* + *Am. parvitarsum*, and a clade with the remaining *Amblyomma* species.

### Origin of diversification and biogeographical history

Within Ixodidae, the recent incorporation in molecular phylogenetic analyses of *Africaniella transversale* and some species assigned previously to “*Aponomma*” have demonstrated that the morphological diversity of *Amblyomma *sensu stricto is due to the retention of plesiomorphic characters that mislead the circumscription of its living fauna [13–15] and potentially those of extinct fauna [68]. Thus, with the aim to evaluate an independent hypothesis of the origin and diversification of *Amblyomma* that is not linked to a potential bias related to the assignation of non-phylogenetically corroborated lineages, we used geographic dating from well-established phylogenetic frameworks of the *Am. cajennense* complex [20, 28] and *Am. parvum* [22] as an alternative to the taxonomic and phylogenetic unresolved questions of the genus.

The higher diversity of *Amblyomma* is displayed in the Neotropic region, which constitutes a biogeographic ecoregion extending from the center of Mexico to the southernmost point of South America, including the Caribbean Islands [69]. This region has a geological and paleoclimatic complex history given that mountain ranges with different origins converge, and several climatic fluctuations plus associated glaciations have occurred [70]. These circumstances have given rise to intricate scenarios that could explain the hyper diversity of the Neotropical region [71]. Additionally, this diversity could have been boosted by geological events through time, including: (i) continental drift [72, 73]; (ii) the intermittent connection through bridges, such as the Antarctic bridge that connected the southern cone of South America with the Australian Region [74, 75], and the Berigian bridge with a northern connection to the Palearctic Region [76]; and (iii) the complex geological dynamics in the northern Neotropical such as, for example, formation of the Isthmus of Tehuantepec and Panama [77–79].

Our new reconstructed time-calibrated tree dated the origin of *Amblyomma* at about 47.8 Mya, with a relatively short credibility interval (95% highest posterior density [HPD]: 57–39.6 Mya). This is the youngest origin estimated for *Amblyomma* so far, which controverts older estimations for the origin of the genus at 91 Mya [24], between the late Jurassic and Early Cretaceous [80], or at 89–77 Mya [23], as well of the Burmese amber fossils assigned to the genus (at 100 Mya [68, 81]). The inconsistencies of our results with previous works could be related to the representativeness of taxa and exclusive analyses of the mt genome. The absence of fossil calibrations may also result in an underestimation of divergence dates. Further work is needed to evaluate the time divergences of *Amblyomma* using solid topologies, perhaps including nuclear more information and calibrations of endemic species of islands (e.g., Galapagos, Hispaniola).

**Fig. 2:**
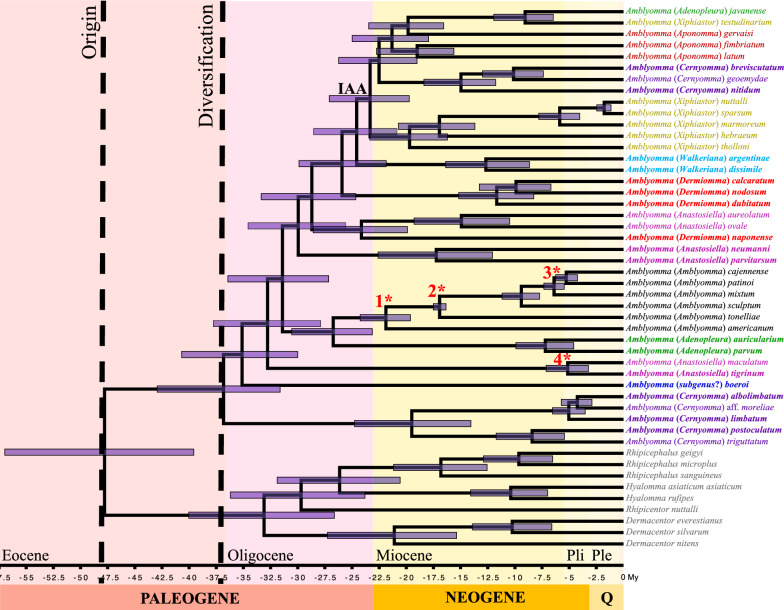
Chronogram with age estimates of major divergence events among *Amblyomma* spp., based on the mitogenomic data set using Bayesian relaxed dating methods (BEAST) and topology 2 (Additional file 1: Fig. S1b). Horizontal bars represent 95% credibility intervals of relevant nodes, and the four calibration constraints are indicated in red with a number and asterisk on the corresponding nodes following the corresponding order date of the text. Dates (and credibility intervals) are in millions of years. Origin and diversification of *Amblyomma* are highlighted with a vertical broken line. Periods on the geological table are shown in different colors. The specimens sequenced in this study are highlighted in bold font. Species with the same subgeneric status are indicated in the same color. IAA, lineage with Indomalayan, Afrotropical, and Australasian distributions; My, million years; Ple, Pleistocene; Pli, Plicene; Q, Quaternary

The origin of diversification of *Amblyomma* was estimated to have occurred 36.8 Mya (95% HPD: 43–31.6 Mya), an age that matches with the end of the Antarctic bridge connection of the southern hemisphere, in the Late Eocene, at about 35 Mya [74, 75]. The origin age of the diversification of the genus has been estimated at 74–60 Mya by Seabolt [23], which is close to the start of continental connections of the southern hemisphere at 65 Mya [74, 75]. Both scenarios do not rule out a Southern Hemisphere fauna connection. Additionally, our time-calibrated tree suggests an origin and posterior diversification of IAA lineages (positioned derivatives in Figs. 1, 2) between 24.6 and 23.3 Mya (95% HPD: 28.5–19.7 Mya).

**Fig. 3:**
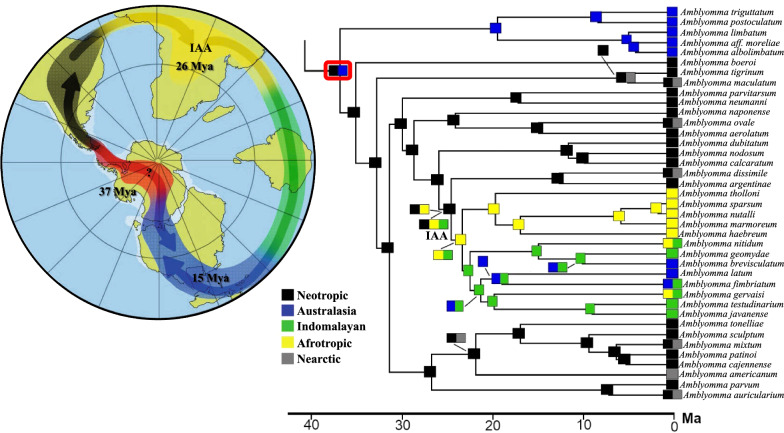
Summary of the ancestral area reconstructions based on the time-tree calibrated (Fig. 2) with the five models used. Five stages were assessed, each of which is indicated with a different color. IAA indicates the lineage with Indomalayan, Afrotropical, and Australasian distributions. A graphic interpretation of the diversification patterns is included

We reconstructed the biogeographic patterns of *Amblyomma* with the time divergence framework. The ancestral area reconstructions under each evaluated biogeographical model were highly consistent (Fig. 3; Additional file 7: Fig. S3a–d; Additional file 4: Table S5). Nevertheless, models with the founder-event speciation parameter (j parameter, alternative hypothesis) outperformed those without it (null hypothesis) (DEC vs DEC + J,* P* = 0.0011; DIVALIKE vs DIVALIKE + J,* p* = 0.007). According to the log-likelihood (LnL) and AICc values, the DEC + J model was the best selected model (Additional file 4: Table S5) and, therefore, we have focused on reporting the results under this model. Our best biogeographic scenario supports the origin of the diversification of *Amblyomma* in the southern hemisphere at the end of the Eocene (Fig. 3), potentially associated with the faunistic flow in the final Antarctic Bridge connection [74, 75]. This scenario agrees with the South American and Australian lineages (here well represented) as the early diverging event in *Amblyomma* (Fig. 1) reached with high statistical support. This does not discard the hypothesis which argues that the origin of *Amblyomma* is in the Neotropic with a posterior colonization to Australia [24]. However, a solid phylogenomic framework of Metastriate (including main lineages of all genera) is necessary to evaluate the biogeographic patterns and ancestral distribution of the related lineages to *Amblyomma*, which could explain the Burmese amber fossil with a widespread ancestral distribution of the closest ancestors of *Amblyomma* [23, 68, 81]. Likewise, this could also make sense of an older origin of diversification of other lineages restricted to the early divergences of Metastriata, which have a notable Gondwanan distribution, as is the case of *Archaeocroton sphenodonti* (New Zealand), *Robertsicus elaphensis* (Nearctic), *Bothriocroton* (Australia) and *Africaniella transversale* (Afrotropic) [24].

While it is necessary to increase the representation of *Amblyomma* species in a phylogenomic framework, the biogeographic hypotheses proposed here support independent expansions along the Neocene to Quaternary era, as is the case of *Am. dissimile*, *Am. ovale* and *Am. auricularium* lineages. Also, our scenario supports a colonization event of *Am. americanum* and its related species (Fig. 3), despite these are not included in the present study.

### Conclusions

Phylogenetic trees from mt genomes (Fig. 4 in [25]; [16]), nuclear rRNA (e.g. Fig. 5 in [16]; [26]; Fig. 8 in [27]), and from combinations of mt and nuclear rRNA genes and morphology [82] have hinted that the Australasian *Amblyomma* or the Australasian *Amblyomma* plus the *Amblyomma* species from South America might be the sister-group to the *Amblyomma* in the rest of the world. In the present study, however, we found the strongest evidence yet that Australasian *Amblyomma* may indeed be the sister-group to the *Amblyomma* of the rest of the world (Figs. 1, 3). The position of the Argentinian tick *Amblyomma boeroi *hints that the most recent common ancestor to the *Amblyomma* evolved in a region between South America and Australia, which would concur with the out-of-Antarctica hypothesis [24, 83]. Thus, on the one hand, the ancestor of the Australasian *Amblyomma* dispersed from Antarctica into that part of Gondwana which became Australasia, and on the other hand, the ancestor of the South American *Amblyomma* dispersed from Antarctic into that part of Gondwana which became South America. Our tree hypothesis also reveals that all *Amblyomma* subgenera included in our study, with the exception of *Walkeriana* and *Amblyomma*, are not monophyletic. Our findings suggest an origin of *Amblyomma* and its posterior diversification to be more recent than the previous hypotheses (47.8 and 36.8 Mya, respectively). Also, the biogeographic analyses reveal the colonization patterns of some neotropical *Amblyomma* species to the Nearctic. Finally, it has been well documented [84, 85] that increasing taxon sampling has a positive effect on tree reconstruction, resulting in more accurate topologies. Thus, future work should aim to reconstruct phylogenetic trees with a denser taxon sampling across both *Amblyomma* and Metastriate.

### Supplementary Information

**Additional file 1: Table S1.** Complete list of the specimens used in this study with the respective GenBank accession number (#), localities, mt genome length, author reference and molecular strategy used. Asterisk indicates partial mt genomes.

**Additional file 2: Table S2.** Amplification strategy for mt genomes. The gene order of the mt genomes are indicated, where a gray color indicates the amplified and sequenced fragment of each species. The general and specific primers used are highlighted in yellow and red, respectively. The sequence of each primer is displayed in the 5’ to 3’ direction.

**Additional file 3: Table S3.** Dataset features, genetic codification, taxa included, best-fit partition schemes and models and log-likelihood (Ln likelihood) values of all phylogenetic analyses.

**Additional file 4: Table S4.** Presence (1) and absence (0) of the species included in the five biogeographic ecoregions assessed: Nearctic, Neotropic, Austral, Afrotropic, Indomalayan. **Table S5.** Report of statistical results across the four models implemented in BioGeoBear, including log-likelihood (LnL), number of parameters, f, e, j parameters and the Akaike information criterion (AICc) values.

**Additional file 5: Figure S1. a** Phylogenetic tree based on mt genome data: Topology 1 with 15 concatenated genes at nucleotide codification (protein-coding and ribosomal genes). Bayesian tree was reconstructed using the best fit evolutionary model and partition scheme. Numbers at nodes are statistical support values for bootstrap proportions. The scale bar is in expected substitutions/site. **b** Phylogenetic tree based on mt genome data: Topology 2 with 15 concatenated genes at nucleotide codification (protein-coding and ribosomal genes) implemented GBlock to trim the alignment. Bayesian tree was reconstructed using the best fit evolutionary model and partition scheme. Numbers at nodes are statistical support values for posterior probability. The scale bar is in expected substitutions/site. **c** Phylogenetic tree based on mt genome data: Topology 3 with 15 concatenated genes at nucleotide codification (protein-coding and ribosomal genes). Maximum-Likelihood tree was reconstructed using the best fit evolutionary model and partition scheme. Numbers at nodes are statistical support values for bootstrap proportions. The scale bar is in expected substitutions/site.** d** Phylogenetic tree based on mt genome data: Topology 4 with 15 concatenated genes at nucleotide codification (protein-coding and ribosomal genes) implemented GBlock to trim the alignment. Maximum-likelihood tree was reconstructed using the best fit evolutionary model and partition scheme. Numbers at nodes are statistical support values for bootstrap proportions. The scale bar is in expected substitutions/site.** e** Phylogenetic tree based on mt genome data: Topology 5 with 15 concatenated genes at nucleotide codification (protein-coding and ribosomal genes). Maximum-likelihood tree was reconstructed using the best fit evolutionary model for the concatenated matrix. Numbers at nodes are statistical support values for bootstrap proportions. The scale bar is in expected substitutions/site.** f** Phylogenetic tree based on mt genome data: Topology 6 with 15 concatenated genes at nucleotide codification (protein-coding and ribosomal genes) implemented GBlock to trim the alignment. Maximum-likelihood tree was reconstructed using the best fit evolutionary model for the concatenated matrix. Numbers at nodes are statistical support values for bootstrap proportions. The scale bar is in expected substitutions/site.** g** Phylogenetic tree based on mt genome data: Topology 7 with 13 concatenated genes at amino acid codification (protein-coding genes). Maximum-likelihood tree was reconstructed using the best fit evolutionary model for the concatenated matrix. Numbers at nodes are statistical support values for bootstrap proportions. The scale bar is in expected substitutions/site.** h** Phylogenetic tree based on mt genome data: Topology 8 with 13 concatenated genes at amino acid codification (protein-coding genes) implemented GBlock to trim the alignment. Maximum-likelihood tree was reconstructed using the best fit evolutionary model for the concatenated matrix. Numbers at nodes are statistical support values for bootstrap proportions. The scale bar is in expected substitutions/site.** i** Phylogenetic tree based on mt genome data: Topology 9 with 13 concatenated genes at amino acid codification (protein-coding genes). Bayesian tree was reconstructed using CAT-GTR evolutionary model for concatenated matrix. Numbers at nodes are statistical support values for bootstrap proportions. The scale bar is in expected substitutions/site.** j** Phylogenetic tree based on mt genome data: Topology 10 with 13 concatenated genes at amino acid codification (protein-coding genes) implemented GBlock to trim the alignment. Bayesian tree was reconstructed using CAT-GTR evolutionary model for concatenated matrix. Numbers at nodes are statistical support values for bootstrap proportions. The scale bar is in expected substitutions/site.** k** Phylogenetic tree based on mt genome data: Topology 11 with 13 concatenated genes at amino acid codification (protein-coding genes). Maximum-likelihood tree was reconstructed using mixture evolutionary model for concatenated matrix. Numbers at nodes are statistical support values for bootstrap proportions. The scale bar is in expected substitutions/site.** l** Phylogenetic tree based on mt genome data: Topology 12 with 13 concatenated genes at amino acid codification (protein-coding genes) implemented GBlock to trim the alignment. Maximum-likelihood tree was reconstructed using mixture evolutionary model for concatenated matrix. Numbers at nodes are statistical support values for bootstrap proportions. The scale bar is in expected substitutions/site.

**Additional file 6: Figure S2.** Phylogenetic tree based on nuclear ribosomal cluster. Maximum-Likelihood tree was reconstructed using the best fit evolutionary model for the concatenated matrix. Numbers at nodes are statistical support values for bootstrap proportions. The scale bar is in expected substitutions/site. The specimens sequenced in this study are highlighted in bold font with their respective GenBank access number in parentheses.

**Additional file 7: Figure S3.** **a** Biogeographic hypothesis using Biogeographic hypothesis using BioGeoBEARS DEC on garrapatas M0_unconstrained ancstates: global optim, 5 areas max. d = 0.0024; e = 0; j = 0; LnL = − 71.43. **b** Biogeographic hypothesis using BioGeoBEARS DEC+J on garrapatas M0_unconstrained ancstates: global optim, 5 areas max. d = 0.0018; e = 0; j = 0.0116; LnL = − 66.07. **c** Biogeographic hypothesis using BioGeoBEARS DIVALIKE on Psychotria M0_unconstrained ancstates: global optim, 5 areas max. d = 0.0028; e = 0; j = 0; LnL = − 69.79. ** d** Biogeographic hypothesis using BioGeoBEARS DIVALIKE+J on Psychotria M0_unconstrained ancstates: global optim, 5 areas max. d = 0.0019; e = 0; j = 0.0104; LnL = − 66.16. Note: In all scenarios (**a**–**d**) five stages were assessed, each of them indicated with its respective abbreviation as follows: Nearctic, NE; Neotropic, NO; Australia, AU; Afrotropic, AF; Indomalayan, IN.

## Data Availability

GenBank accession numbers generated in this study are available in Additional file 1: Table S1 and Additional file 7: Fig. S2.
