# Supplementary material for: New insights into the molecular phylogeny, biogeographical history, and diversification of Amblyomma ticks (Acari: Ixodidae) based on mitogenomes and nuclear sequences
Source: Parasit Vectors. 2024 Mar 18;17:139. doi: 10.1186/s13071-024-06131-w (PMC10946108; doi:10.1186/s13071-024-06131-w)
Supplement: Supplementary file 1 — Additional file 1: Table S1. Complete list of the specimens used in this study with the respective GenBank accession number (#), localities, mt genome length, author reference and molecular strategy used. Asterisk indicates partial mt genomes. [file 13071_2024_6131_MOESM1_ESM.docx]

Additional file 1: Table S1. Complete list of the specimens used in this study with the respective GenBank accession number (#), localities, mt genome length, author reference, and molecular strategy used. With asterisk are indicated the partial mt genomes.

| **Genbank #, ID** | ***Genus*** | ***Subgenus*** | ***Species*** | **Locality** | **Lenght** | **Reference** |
| --- | --- | --- | --- | --- | --- | --- |
| OR899809, AM12 | *Amblyomma* | *Anastosiella* | *tigrinum* | Quilino, Córdoba, Argentina | 14808 | This study (amplicons) |
| OR899815, AM15 | *Amblyomma* | *Adenopleura* | *auricularium** | Puerto Carreño, Vichada, Colombia | partial | This study (amplicons) |
| OR899814, SA16 | *Amblyomma* | *Dermiomma* | *calcaratum** | Km 41, Conexión Pacifico 3 Highway, Manizales Colombia | partial | This study (amplicons) |
| OR899813, SA17 | *Amblyomma* | *Dermiomma* | *nodosum** | Km 41, Conexión Pacifico 3 Highway, Manizales Colombia | partial | This study (amplicons) |
| OR899804, T04 | *Amblyomma* | *Walkeriana* | *dissimile* | Universidad del Magdalena, Santa Marta, Colombia | 14727 | This study (amplicons) |
| OR899807, AM02 | *amblyomma* | *Dermiomma* | *naponense* | Panama | 14808 | This study (WGS) |
| OR899812, AM03 | *Amblyomma* | *Adenopleura* | *parvum* | Quilino, Córdoba, Argentina | 14611 | This study (WGS) |
| OR899811, AM04NEU | *Amblyomma* | *Anastosiella* | *neumanni* | Dean Funes, Córdoba, Argentina | 14865 | This study (WGS) |
| OR899806, AM06 | *Amblyomma* |  | *boeroi* | Rivadavia Banda Sur, Salta, Argentina | 14678 | This study (WGS) |
| OR899810, AM07 | *Amblyomma* | *Anastosiella* | *parvitarsum* | San Antonio de los Cobres, Salta, Argentina | 14875 | This study (WGS) |
| OR899805, AM08 | *Amblyomma* | *Walkeriana* | *argentinae* | Colonia Dora, Santiago del Estero, Argentina | 14716 | This study (WGS) |
| OR899808, AM09 | *Amblyomma* | *Dermiomma* | *dubitatum* | Romang, Santa Fe, Argentina | 14750 | This study (WGS) |
| OR350526, AN26 | *Amblyomma* | *Cernyomma* | *nitidum* | Okinawa, Japan | 14757 | This study (WGS) |
| OR416214, AU071-B5296 | *Amblyomma* | *Cernyomma* | *breviscutatum* | Innisfail, Queensland, Australia | 14762 | This study (WGS) |
| OR350527, B5475a | *Amblyomma* | *Cernyomma* | *postoculatum* | Hall Creek, Western Australia, Australia | 14751 | This study (WGS) |
| OR350524, B5607 | *Amblyomma* | *Cernyomma* | *albolimbatum* | Middleback Range, South Australia, Australia | 14834 | This study (WGS) |
| OR416215, B6548 | *Amblyomma* | *Cernyomma* | *limbatum* | Maude, South Australia, Australia | 14823 | This study (WGS) |
| OP901707 | *Amblyomma* | *Amblyomma* | *cajennense* | Mato Grosso, Brazil | 14774 | [1] |
| OP901706 | *Amblyomma* | *Amblyomma* | *sculptum* | Formosa, Argentina | 14784 | [1] |
| OP901704 | *Amblyomma* | *Amblyomma* | *patinoi* | Villeta, Colombia | 14779 | [1] |
| OP901702 | *Amblyomma* | *Amblyomma* | *mixtum* | Santa Marta, Colombia | 14815 | [1] |
| OP901705 | *Amblyomma* | *Amblyomma* | *tonelliae* | Parque Nacional Copo, Santiago del Estero, Argentina | 14731 | [1] |
| OL741734 | *Amblyomma* | *Aponomma* | *gervaisi* | Imported to Japan from Sri Lanka | 14709 | [2] |
| OL741735 | *Amblyomma* | *Aponomma* | *latum* | Imported to Japan from Ghana | 14658 | [2] |
| OQ842962 | *Amblyomma* | *Xiphiastor* | *sparsum* | Southwest Senegal | 14679 | [3] |
| OL741736 | *Amblyomma* | *Xiphiastor* | *nuttalli* | Imported to Japan from Zambia | 14681 | [2] |
| KY457515 | *Amblyomma* | *Xiphiastor* | *marmoreum* | Irene, South Africa | 14676 | [4] |
| KY457513 | *Amblyomma* | *Xiphiastor* | *hebraeum* | Soutpan, South Africa | 14654 | [4] |
| KY457522 | *Amblyomma* | *Xiphiastor* | *tholloni* | Kruger National Park, South Africa | 14640 | [4] |
| MK814531 | *Amblyomma* | *Cernyomma* | *geoemydae* | Nanning City, Guangxi Province, China | 14780 | [5] |
| MT029329 | *Amblyomma* | *Xiphiastor* | *testudinarium* | Nanning City, Guangxi Province, China | 14760 | [6] |
| MW719251 | *Amblyomma* | *Anastosiella* | *maculatum* | Oklahoma State University Tick Rearing Facility, USA | 14803 | [7] |
| NC_005963 | *Amblyomma* | *Cernyomma* | *triguttatum* | No provides | 14740 | [8] |
| NC_017759 | *Amblyomma* | *Aponomma* | *fimbriatum* | Lizard Island, Australia | 14705 | [9] |
| NC_027609 | *Amblyomma* | *Amblyomma* | *americanum* | Panola Mountain State Park near Atlanta, Georgia, USA | 14709 | [10] |
| NC_043872 | *Amblyomma* | *Adenopleura* | *javanense* | No provides | 14780 | [11] |
| NC_050255 | *Amblyomma* | *Anastosiella* | *ovale* | Reserva El Bagual, Formosa Province, Argentina | 14760 | [12] |
| SRR4301110 | *Amblyomma* | *Anastosiella* | *aureolatum** | Atibaia strain, Sao Paulo, Brazil | partial | [13] (RNA-seq) |
| SRR8074777 | *Amblyomma* | *Cernyomma* | *aff. moreliae** | Sydney, Australia | partial | [14] (RNA-seq) |
| NC_042764 | *Dermacentor* |  | *everestianus* | North Lhasa City, Tibet Autonomous Region, China | 15191 | [15] |
| NC_023349 | *Dermacentor* |  | *nitens* | Campo Grande, Brazil | 14839 | [16] |
| NC_026552 | *Dermacentor* |  | *silvarum* | Wanda mountain, Heilongjiang, China | 14945 | [17] |
| MF101817 | *Hyalomma* |  | *asiaticum asiaticum* | Qitai County, Xinjiang Uygur Autonomous Region, China | 14720 | [18] |
| MW884229 | *Hyalomma* |  | *rufipes* | China | 14761 | [19] |
| NC_023350 | *Rhipicephalus* |  | *geigyi* | Burkina Faso | 14948 | [16] |
| NC_023335 | *Rhipicephalus* |  | *microplus* | Mato Grosso do Sul, Brazil | 14905 | [16] |
| NC_002074 | *Rhipicephalus* |  | *sanguineus* | No provides | 14710 | [20] |
| NC_039828 | *Rhipicentor* |  | *nuttalli* | No provides | 14779 | Mans et al. [Unpublished] |
| NC_041076 | *Haemaphysalis* |  | *bancrofti* | Australia | 14673 | Burnard et al., [Unpublished] |
| NC_020334 | *Haemaphysalis* |  | *formosensis* | Kabutoyama, Nishinomiya, Hyogo, Japan | 14676 | [21] |
| NC_039765 | *Haemaphysalis* |  | *hystricis* | Qiliping Town, Hong’an County, Hubei Province, China | 14716 | [22] |
| NC_037246 | *Haemaphysalis* |  | *japonica* | Fangshan district, Beijing, China | 14685 | [23] |
| NC_020335 | *Haemaphysalis* |  | *inermis* | Western Romania | 14846 | [21] |
| NC_017756 | *Bothriocroton* |  | *concolor* | Kangaroo Island, SA, Australia | 14809 | [9] |
| NC_017757 | *Bothriocroton* |  | *undatum* | Fraser Island, QLD, Australia | 14769 | [9] |
| NC_017745 | *Archaeocroton* |  | *sphenodonti* | Stephens Island, New Zealand | 14772 | [9] |
| NC_017758 | *Robertsicus* |  | *elaphensis* | Texas, USA | 14627 | [9] |
| NC_002010 | *Ixodes* |  | *hexagonus* | No provides | 14539 | [24] |
| NC_018369 | *Ixodes* |  | *ricinus* | Monte Bollettone, Italy | 14566 | [25] |
| NC_041086 | *Ixodes* |  | *tasmani* | Australia | 15227 | [26] |

References

1. Cotes-Perdomo AP, Nava S, Castro LR, Rivera-Paéz FA, Cortés-Vecino JA, Uribe JE. Phylogenetic relationships of the *Amblyomma cajennense* complex (Acari: Ixodidae) at mitogenomic resolution. Ticks Tick-borne Dis. 2023;14(3):102125.
2. Kelava S, Mans BJ, Shao R, Moustafa MAM, Matsuno K, Takano A, et al.. Phylogenies from mitochondrial genomes of 120 species of ticks: Insights into the evolution of the families of ticks and of the genus *Amblyomma*. Ticks Tick-borne Dis. 2021;12(1):101577.
3. Cotes-Perdomo AP. Sánchez-Vialas A, Thomas R, Jenkins Andrew, Uribe JE. New insights into the systematics of the Afrotropical *Amblyomma marmoreum* complex (Acari, Ixodidae) and a novel *Rickettsia africae* strain using morphological and metagenomic approaches. bioRxiv. 2023;08.
4. Mans BJ, Featherson J, Kvas M, Pillay KA, de Klerk D, Pienaar R, et la. Argasid and ixodid systematics: implications for soft tick evolution and systematics, with a new argasid species list. Ticks Tick-borne Dis. 2019;10(1):219-240.
5. Chang QC, Hu Y, Que TC, Liu YX, Zhu JG, Diao. The complete mitochondrial genome of *Amblyomma geoemydae* (Ixodida: Ixodidae). Mitochondrial DNA B: Resour. 2019;4(2):2551-2552.
6. Chang QC, Wu TT, Cao H, Sun MQ, Zhang WT, Xue SJ. The complete mitochondrial genome of *Amblyomma testudinarium* (Ixodida: Ixodidae). Mitochondrial DNA B: Resour. 2020;5(2):1485-1486.
7. Brenner AE, Raghavan R. Complete Mitochondrial Genome Sequence of the Gulf Coast Tick (Amblyomma maculatum). Microbiol. Resour. Announc. 2021;10(38):e00431-21.
8. Shao R, Fukunaga M, Barker SC. The mitochondrial genomes of ticks and their kin: a review plus the description of the mitochondrial genomes of *Amblyomma triguttatum* and *Ornithodoros porcinus*. In Proceedings of the 5th international conference on TTP7. Université de Neuchâtel Switzerland. 2005.
9. Burger TD, Shao R, Beati L, Miller H, Barker SC. Phylogenetic analysis of ticks (Acari: Ixodida) using mitochondrial genomes and nuclear rRNA genes indicates that the genus *Amblyomma* is polyphyletic. Mol. Phylogenetics Evol. 2012;64(1):45-55.
10. Williams-Newkirk AJ, Burroughs M, Changayil SS, Dasch GA. The mitochondrial genome of the lone star tick (Amblyomma americanum). Ticks Tick-borne Dis. 2015;6(6):793-801.
11. Duan DY, Tang JM, Chen Z, Liu GH, Cheng TY. (2020). Mitochondrial genome of *Amblyomma javanense*: a hard tick parasite of the endangered Malayan pangolin (*Manis javanica*). Med. Vet. Entomol. 2020;34(2):229–235.
12. Uribe JE, Nava S, Murphy KR, Tarragona EL, Castro LR. Characterization of the complete mitochondrial genome of *Amblyomma ovale*, comparative analyses and phylogenetic considerations. Exp. Appl. Acarol. 2020;81(3):421–439.
13. Martins LA, Galletti MFDM, Ribeiro JM, Fujita A, Costa FB, Labruna MB, et al. The distinct transcriptional response of the midgut of *Amblyomma sculptum* and *Amblyomma aureolatum* ticks to *Rickettsia rickettsii* correlates to their differences in susceptibility to infection. Front. cell. infect. 2017;7:129.
14. Harvey E, Rose K, Eden JS, Lo N, Abeyasuriya T, Shi M, et al. Extensive diversity of RNA viruses in Australian ticks. Virol. J. 2019;93(3):e01358–18.
15. Yu Z, Zhang S, Wang T, Yang X, Wang H, Liu J. The mitochondrial genome and phylogenetic analysis of the tick *Dermacentor everestianus* Hirst, 1926 (Acari: Ixodidae). Systematic and Applied Acarology, 2018;23(7):1313–1321.
16. Burger TD, Shao R, Barker SC. Phylogenetic analysis of mitochondrial genome sequences indicates that the cattle tick, *Rhipicephalus* (*Boophilus*) *microplus*, contains a cryptic species. Mol. Phylogenetics Evol. 2014;76:241-253.
17. Guo DH, Zhang Y, Fu X, Gao Y, Liu YT, Qiu JH, et al. Complete mitochondrial genomes of *Dermacentor silvarum* and comparative analyses with another hard tick *Dermacentor nitens*. Exp. Parasitol. 2016;169:22–27.
18. Liu ZQ, Liu YF, Kuermanali N, Wang DF, Chen SJ, Guo HL, et al. Sequencing of complete mitochondrial genomes confirms synonymization of *Hyalomma asiaticum asiaticum* and kozlovi, and advances phylogenetic hypotheses for the Ixodidae. PloS one, 2018;13(5):e0197524.
19. Lang J, Shan Y, Zhang M, Liu J, Wang F. The complete mitochondrial genome of *Hyalomma rufipes* (Acari: Ixodidae) from China and comparative analysis of mt genomes in genus *Hyalomma*. Int. J. Acarol. 2022;48(2):87–97.
20. Liu GH, Chen F, Chen YZ, Song HQ, Lin RQ, Zhou DH, Zhu XQ. Complete mitochondrial genome sequence data provides genetic evidence that the brown dog tick *Rhipicephalus sanguineus* (Acari: Ixodidae) represents a species complex. Int. J. Biol. Sci. 2013;9(4):361.
21. Burger TD, Shao R, Barker SC. Phylogenetic analysis of the mitochondrial genomes and nuclear rRNA genes of ticks reveals a deep phylogenetic structure within the genus *Haemaphysalis* and further elucidates the polyphyly of the genus *Amblyomma* with respect to *Amblyomma sphenodonti* and *Amblyomma elaphense*. Ticks Tick-borne Dis. 2013;4(4):265–274.
22. Tian J, Ge M, Xu H, Wu T, Yu B, Lei C. The complete mitochondrial genome and phylogenetic analysis of *Haemaphysalis hystricis* (Parasitiformes: Ixodidae). Mitochondrial DNA B: Resour. 2019;4(1):1049–1050.
23. Chang QC, Diao PW, Fu X, Wang XX, Qiu YY, Hu Y, et al. The complete mitochondrial genome of *Haemaphysalis japonica* (Ixodida: Ixodidae). Mitochondrial DNA B: Resour. 2019;4(1):1006–1007.
24. Black 4th WC, Roehrdanz RL. Mitochondrial gene order is not conserved in arthropods: prostriate and metastriate tick mitochondrial genomes. Mol. Biol. Evol. 1998;15(12):1772–1785.
25. Montagna M, Sassera D, Griggio F, Epis S, Bandi C, Gissi C. Tick-box for 3′-end formation of mitochondrial transcripts in ixodida, basal chelicerates and drosophila. PloS one, 2012;0047538.
26. Burnard D, Shao R. Mitochondrial genome analysis reveals intraspecific variation within Australian hard tick species. Ticks Tick-borne Dis. 2019;10(3):677-681.
