# Supplementary material for: New insights into the molecular phylogeny, biogeographical history, and diversification of Amblyomma ticks (Acari: Ixodidae) based on mitogenomes and nuclear sequences
Source: Parasit Vectors. 2024 Mar 18;17:139. doi: 10.1186/s13071-024-06131-w (PMC10946108; doi:10.1186/s13071-024-06131-w)
Supplement: Supplementary file 2 — Additional file 2: Table S2. Amplification strategy for mt genomes. The gene order of the mt genomes are indicated, where a gray color indicates the amplified and sequenced fragment of each species. The general and specific primers used are highlighted in yellow and red, respectively. The sequence of each primer is displayed in the 5’ to 3’ direction. [file 13071_2024_6131_MOESM2_ESM.docx]

Additional file 2: Table S2. Amplification strategy for mt genomes. The gene order of the mt genomes are indicated, where grey color indicating the amplified and sequenced fragment of each specie. In yellow and red are highlighted the general and specific primers used, respectively. The sequence of each primer is displayed in 5’ to 3’ direction.

| **Genbank #, ID** | ***Genus*** | ***Subgenus*** | ***Species*** |
| --- | --- | --- | --- |
| OR899809, AM12 | *Amblyomma* | *Anastosiella* | *tigrinum* |
| OR899815, AM15 | *Amblyomma* | *Adenopleura* | *auricularium** |
| OR899814, SA16 | *Amblyomma* | *Dermiomma* | *calcaratum** |
| OR899813, SA17 | *Amblyomma* | *Dermiomma* | *nodosum** |
| OR899804, T04 | *Amblyomma* | *Walkeriana* | *dissimile* |
|  |  |  |  |
| Primer | Direction | Primer sequence 5'–3' | Reference |
| Caj1F-Lys | Forward | TTTAAGCRATGGTCTCTTAAACCAA | Cotes-Perdomo et al., 2023 |
| Caj2R-Lys | Reverse | KKTTGGTTTAAGAGACCATYGCTT | Cotes-Perdomo et al., 2023 |
| Caj2F-Arg | Forward | TTCAGTTTCGGCCTGAATTTAGAA | Cotes-Perdomo et al., 2023 |
| Caj2R-Arg | Reverse | TTCTAAATTCAGGCCGAAACTGAA | Cotes-Perdomo et al., 2023 |
| Caj3F-Ile | Forward | GTCCAGTAAATGHGATAGCCGGTTG | Cotes-Perdomo et al., 2023 |
| Caj3R-Ile | Reverse | CAACCGGCTATCDCATTTACTGGAC | Cotes-Perdomo et al., 2023 |
| Caj4F-cytb | Forward | CATATTCAACGAGATGTAAATTATGG | Cotes-Perdomo et al., 2023 |
| Caj4R-cytb | Reverse | CCATAATTTACATCTCGTTGAATATG | Cotes-Perdomo et al., 2023 |
| Amtig-rrnSR | Reverse | AATGAGAGCGACGGGCGATGT | Beati and Keirans, 2001 |
| Amcal-cox1F | Forward | ATGGTCCCTCAGTTGACATAGCTAT | This study |
| Amdis-cox1F | Forward | TTGGAAGATGATCTGGAATTCTAGG | This study |
| Amdis-cox1R | Reverse | GGGAAATGCTATATCTGGAGCTCC | This study |
| TK-16S-F1-rrnL | Forward | GTTTGCGACCTCGATGTTGGATTAGG | Uribe et al., 2020 |
| TK-16S-R-rrnL | Reverse | CGGTYTRAACTCAGATCATGTAGG | Uribe et al., 2020 |
