# Supplementary material for: New insights into the molecular phylogeny, biogeographical history, and diversification of Amblyomma ticks (Acari: Ixodidae) based on mitogenomes and nuclear sequences
Source: Parasit Vectors. 2024 Mar 18;17:139. doi: 10.1186/s13071-024-06131-w (PMC10946108; doi:10.1186/s13071-024-06131-w)
Supplement: Supplementary file 3 — Additional file 3: Table S3. Dataset features, genetic codification, taxa included, best-fit partition schemes and models and log-likelihood (Ln likelihood) values of all phylogenetic analyses. [file 13071_2024_6131_MOESM3_ESM.docx]

Additional file 3: Table S3. Data set features, genetic codification, taxa included, best-fit partition schemes and models, and log-likelihood *(– Ln* likelihood) values of all phylogenetic analyses.

| # | **Matrix** | **Codification** | **No. Taxa** | **Inference** | **Sites** | ***– Ln* likelihood** | **Partitions and/or models** |
| --- | --- | --- | --- | --- | --- | --- | --- |
| 1 | BI-NT-Partitioned | NT | 60 | BI | 13141 | -35.27509 | 1 [GTR+I+G] = 1-837\3 10900-11716 11717-13141; 2 [GTR+I+G] = 2-837\3 838-2379\3 839-2379\3 2381-3054\3 3056-3837\3 3839-4914\3 4916-5859\3 5861-6831\3 6832-7173\3 6833-7173\3 7175-8787\3 8789-10458\3 10460-10899\3; 3 [GTR+I+G] = 3-837\3 3057-3837\3 4917-5859\3 5862-6831\3 6834-7173\3 7176-8787\3 8790-10458\3 10461-10899\3; 4 [GTR+I+G] = 840-2379\3 2382-3054\3 3840-4914\3; 5 [GTR+I+G] = 2380-3054\3 3055-3837\3 3838-4914\3 4915-5859\3 5860-6831\3 7174-8787\3 8788-10458\3 10459-10899\3; |
| 2 | BI-NT-Partitioned-GB | NT | 60 | BI | 11480 | -29.12586 | 1 [GTR+I+G] = 1-684\3 2221-2886\3 2887-3654\3 3655-4704\3 4705-5604\3 5605-6411\3 6412-6729\3 6413-6729\3 6730-8208\3 8209-9717\3 9718-10065\3 10066-10602 10603-11480; 2 [GTR+I+G] = 2-684\3 685-2220\3 686-2220\3 2222-2886\3 2888-3654\3 3656-4704\3 4706-5604\3 5606-6411\3 6731-8208\3 8210-9717\3 9719-10065\3 9720-10065\3; 3 [GTR+I+G] = 3-684\3 687-2220\3 2223-2886\3 5607-6411\3; 4 [GTR+I+G] = 2889-3654\3 3657-4704\3 4707-5604\3 6414-6729\3 6732-8208\3 8211-9717\3; |
| 3 | ML-NT-Partitioned | NT | 60 | ML | 13141 | -35.08150 | 1 [GTR+F+R6] = 1-837\3 10900-11716 11717-13141; 2 [TVM+F+R5] = 2-837\3 838-2379\3 839-2379\3 2381-3054\3 3056-3837\3 3839-4914\3 4916-5859\3 5861-6831\3 6832-7173\3 6833-7173\3 7175-8787\3 8789-10458\3 10460-10899\3; 3 [TIM+F+R6] = 3-837\3 3057-3837\3 4917-5859\3 5862-6831\3 6834-7173\3 7176-8787\3 8790-10458\3 10461-10899\3; 4 [TIM2+F+R5] = 840-2379\3 2382-3054\3 3840-4914\3; 5 [GTR+F+R5] = 2380-3054\3 3055-3837\3 3838-4914\3 4915-5859\3 5860-6831\3 7174-8787\3 8788-10458\3 10459-10899\3; |
| 4 | ML-NT-GBlock-Partitioned | NT | 60 | ML | 11480 | -28.57338 | 1 [GTR+F+R5] = 1-684\3 2221-2886\3 2887-3654\3 3655-4704\3 4705-5604\3 5605-6411\3 6412-6729\3 6413-6729\3 6730-8208\3 8209-9717\3 9718-10065\3 10066-10602 10603-11480; 2 [TVM+F+R5] = 2-684\3 685-2220\3 686-2220\3 2222-2886\3 2888-3654\3 3656-4704\3 4706-5604\3 5606-6411\3 6731-8208\3 8210-9717\3 9719-10065\3 9710-10065\3; 3 [TPM2+F+R6] = 3-684\3 687-2220\3 2223-2886\3 5607-6411\3; 4 [K3Pu+F+R5] = 2889-3654\3 3657-4704\3 4707-5604\3 6414-6729\3 6732-8208\3 8211-9717\3; |
| 5 | ML-NT-TESTNEW | NT | 60 | ML | 13141 | -36.18817 | GTR+F+R6 |
| 6 | ML-NT-GBlock-TESTNEW | NT | 60 | ML | 11480 | -29.91708 | GTR+F+R6 |
| 7 | ML-AA-TESTNEW | AA | 60 | ML | 3801 | -14.21868 | mtMet+F+R6 |
| 8 | ML-AA-GBlock-TESTNEW | AA | 60 | ML | 3619 | -14.69770 | mtMet+F+R7 |
| 9 | BI-AA-CATGTR | AA | 60 | BI | 3801 | 15,13100 \| -13,87900 | CATGTR |
| 10 | BI-AA-GBlock-CATGTR | AA | 60 | BI | 3619 | 14,78700 \| 14,84100 | CATGTR |
| 11 | ML-AA-mixtureM | AA | 60 | ML | 3801 | -14.02876 | mtZOA+C60+F+R7 |
| 12 | ML-AA-GBlock-mixtureM | AA | 60 | ML | 3619 | -14.55431 | mtZOA+C60+F+R7 |
| 13 | TEMETREE-NT | NT | 48 | BEAST | 11480 | Non | 1 [GTR+I+G]= 1-684\3 2887-3654\3 3655-4704\3 4705-5604\3 5605-6411\3 6412-6729\3 6413-6729\3 6730-8208\3 8209-9717\3 9718-10065\3 10066-10602 10603-11480; 2 [GTR+I+G]= 2-684\3 685-2220\3 686-2220\3 2221-2886\3 2222-2886\3 2888-3654\3 3656-4704\3 4706-5604\3 5606-6411\3 6731-8208\3 8210-9717\3 9719-10065\3 9710-10065\3; 3 [GTR+I+G]= 3-684\3 2889-3654\3 4707-5604\3 6414-6729\3 6732-8208\3 8211-9717\3; 4 [GTR+I+G]= 687-2220\3 2223-2886\3 3657-4704\3 5607-6411\3; |
