# Supplementary material for: New insights into the molecular phylogeny, biogeographical history, and diversification of Amblyomma ticks (Acari: Ixodidae) based on mitogenomes and nuclear sequences
Source: Parasit Vectors. 2024 Mar 18;17:139. doi: 10.1186/s13071-024-06131-w (PMC10946108; doi:10.1186/s13071-024-06131-w)
Supplement: Supplementary file 4 — Additional file 4: Table S4. Presence (1) and absence (0) of the species included in the five biogeographic ecoregions assessed: Nearctic, Neotropic, Austral, Afrotropic, Indomalayan. Table S5. Report of statistical results across the four models implemented in BioGeoBear, including log-likelihood (LnL), number of parameters, f, e, j parameters and the Akaike information criterion (AICc) values. [file 13071_2024_6131_MOESM4_ESM.docx]

Additional file 4: Table S4. Presence (1) and absence (0) of the species included in the five biogeographic ecoregions assessed: Nearctic, Neotropic, Austral, Afrotropic, Indomalayan.

| **48 6 (NE NO AU AF IN O)** | **Nearctic** | **Neotropic** | **Australia** | **Afrotropic** | **Indomalayan** | **Outgroup** |
| --- | --- | --- | --- | --- | --- | --- |
| Amblyomma_Adenopleura_auricularium_AM15 | 1 | 1 | 0 | 0 | 0 | 0 |
| Amblyomma_Adenopleura_parvum_AM03 | 0 | 1 | 0 | 0 | 0 | 0 |
| Amblyomma_Amblyomma_cajennense_T19L5 | 0 | 1 | 0 | 0 | 0 | 0 |
| Amblyomma_Amblyomma_mixtum_T01 | 1 | 1 | 0 | 0 | 0 | 0 |
| Amblyomma_Amblyomma_patinoi_SA20 | 0 | 1 | 0 | 0 | 0 | 0 |
| Amblyomma_Amblyomma_sculptum_brL4 | 0 | 1 | 0 | 0 | 0 | 0 |
| Amblyomma_Amblyomma_tonelliae_T18L6 | 0 | 1 | 0 | 0 | 0 | 0 |
| Amblyomma_Anastosiella_aureolatum_SRR4301110 | 0 | 1 | 0 | 0 | 0 | 0 |
| Amblyomma_Anastosiella_neumanni_AM04NEU | 0 | 1 | 0 | 0 | 0 | 0 |
| Amblyomma_Anastosiella_parvitarsum_AM07 | 0 | 1 | 0 | 0 | 0 | 0 |
| Amblyomma_Anastosiella_tigrinum_AM12 | 0 | 1 | 0 | 0 | 0 | 0 |
| Amblyomma_Aponomma_gervaisi_OL741734 | 0 | 0 | 0 | 1 | 1 | 0 |
| Amblyomma_Aponomma_latum_OL741735 | 0 | 0 | 1 | 0 | 0 | 0 |
| Amblyomma_Cernyomma_albolimbatum_OR350524 | 0 | 0 | 1 | 0 | 0 | 0 |
| Amblyomma_Cernyomma_breviscutatum_OR416214 | 0 | 0 | 1 | 0 | 0 | 0 |
| Amblyomma_Cernyomma_limbatum_OR416215 | 0 | 0 | 1 | 0 | 0 | 0 |
| Amblyomma_Cernyomma_moreliae_SRR8074777 | 0 | 0 | 1 | 0 | 0 | 0 |
| Amblyomma_Cernyomma_nitidum_OR350526 | 0 | 0 | 1 | 0 | 1 | 0 |
| Amblyomma_Cernyomma_postoculatum_OR350527 | 0 | 0 | 1 | 0 | 0 | 0 |
| Amblyomma_Dermiomma_calcaratum_SA16 | 0 | 1 | 0 | 0 | 0 | 0 |
| Amblyomma_Dermiomma_dubitatum_AM09 | 0 | 1 | 0 | 0 | 0 | 0 |
| Amblyomma_Dermiomma_naponense_AM022 | 0 | 1 | 0 | 0 | 0 | 0 |
| Amblyomma_Dermiomma_nodosum_SA17 | 0 | 1 | 0 | 0 | 0 | 0 |
| Amblyomma_subgenus_boeroi_AM06 | 0 | 1 | 0 | 0 | 0 | 0 |
| Amblyomma_Walkeriana_argentinae_AM08_1 | 0 | 1 | 0 | 0 | 0 | 0 |
| Amblyomma_Walkeriana_dissimile_T04 | 1 | 1 | 0 | 0 | 0 | 0 |
| Amblyomma_Xiphiastor_nuttalli_OL741736 | 0 | 0 | 0 | 1 | 0 | 0 |
| Amblyomma_Xiphiastor_sparsum_OQ842962 | 0 | 0 | 0 | 1 | 0 | 0 |
| Amblyomma_Amblyomma_americanum_NC_027609 | 1 | 0 | 0 | 0 | 0 | 0 |
| Amblyomma_Aponomma_fimbriatum_NC_017759 | 0 | 0 | 1 | 0 | 1 | 0 |
| Amblyomma_Cernyomma_geoemydae_MK814531 | 0 | 0 | 0 | 0 | 1 | 0 |
| Amblyomma_Xiphiastor_hebraeum_KY457513 | 0 | 0 | 0 | 1 | 0 | 0 |
| Amblyomma_Adenopleura_javanense_NC_043872 | 0 | 0 | 0 | 0 | 1 | 0 |
| Amblyomma_Anastosiella_maculatum_MW719251 | 1 | 1 | 0 | 0 | 0 | 0 |
| Amblyomma_Xiphiastor_marmoreum_KY457515 | 0 | 0 | 0 | 1 | 0 | 0 |
| Amblyomma_Anastosiella_ovale_NC_050255 | 1 | 1 | 0 | 0 | 0 | 0 |
| Amblyomma_Xiphiastor_testudinarium_MT029329 | 0 | 0 | 0 | 0 | 1 | 0 |
| Amblyomma_Xiphiastor_tholloni_KY457522 | 0 | 0 | 0 | 1 | 0 | 0 |
| Amblyomma_Cernyomma_triguttatum_NC_005963 | 0 | 0 | 1 | 0 | 0 | 0 |
| Dermacentor_everestianus_NC_042764 | 0 | 0 | 0 | 0 | 0 | 1 |
| Dermacentor_nitens_NC_023349 | 0 | 0 | 0 | 0 | 0 | 1 |
| Dermacentor_silvarum_NC_026552 | 0 | 0 | 0 | 0 | 0 | 1 |
| Hyalomma_asiaticum_asiaticum_MF101817 | 0 | 0 | 0 | 0 | 0 | 1 |
| Hyalomma_rufipes_MW884229 | 0 | 0 | 0 | 0 | 0 | 1 |
| Rhipicentor_nuttalli_NC_039828 | 0 | 0 | 0 | 0 | 0 | 1 |
| Rhipicephalus_geigyi_NC_023350 | 0 | 0 | 0 | 0 | 0 | 1 |
| Rhipicephalus_microplus_NC_023335 | 0 | 0 | 0 | 0 | 0 | 1 |
| Rhipicephalus_sanguineus_NC_002074 | 0 | 0 | 0 | 0 | 0 | 1 |

Table S5. Report of statistical results across the four models implemented in BioGeoBear, including log-likelihood (LnL), number of parameters, f, e, j parameters, and the Akaike information criterion (AICc) values.

|  | **LnL** | **Number of parameters** | **d** | **e** | **j** | **AICc** |
| --- | --- | --- | --- | --- | --- | --- |
| **DEC** | -71.43 | 2 | 0.0024 | 1.0e-12 | 0 | 146.9 |
| **DEC+J** | -66.07 | 3 | 0.0018 | 1.0e-12 | 0.012 | 138.7 |
| **DIVALIKE** | -69.79 | 2 | 0.0028 | 1.0e-12 | 0 | 143.9 |
| **DIVALIKE + J** | -66.16 | 3 | 0.0019 | 1.0e-12 | 0.010 | 138.9 |
