# Supplementary material for: New insights into the molecular phylogeny, biogeographical history, and diversification of Amblyomma ticks (Acari: Ixodidae) based on mitogenomes and nuclear sequences
Source: Parasit Vectors. 2024 Mar 18;17:139. doi: 10.1186/s13071-024-06131-w (PMC10946108; doi:10.1186/s13071-024-06131-w)
Supplement: Supplementary file 5 — Additional file 5: Figure S1. a Phylogenetic tree based on mt genome data: Topology 1 with 15 concatenated genes at nucleotide codification (protein-coding and ribosomal genes). Bayesian tree was reconstructed using the best fit evolutionary model and partition scheme. Numbers at nodes are statistical support values for bootstrap proportions. The scale bar is in expected substitutions/site. b Phylogenetic tree based on mt genome data: Topology 2 with 15 concatenated genes at nucleotide codification (protein-coding and ribosomal genes) implemented GBlock to trim the alignment. Bayesian tree was reconstructed using the best fit evolutionary model and partition scheme. Numbers at nodes are statistical support values for posterior probability. The scale bar is in expected substitutions/site. c Phylogenetic tree based on mt genome data: Topology 3 with 15 concatenated genes at nucleotide codification (protein-coding and ribosomal genes). Maximum-Likelihood tree was reconstructed using the best fit evolutionary model and partition scheme. Numbers at nodes are statistical support values for bootstrap proportions. The scale bar is in expected substitutions/site. d Phylogenetic tree based on mt genome data: Topology 4 with 15 concatenated genes at nucleotide codification (protein-coding and ribosomal genes) implemented GBlock to trim the alignment. Maximum-likelihood tree was reconstructed using the best fit evolutionary model and partition scheme. Numbers at nodes are statistical support values for bootstrap proportions. The scale bar is in expected substitutions/site. e Phylogenetic tree based on mt genome data: Topology 5 with 15 concatenated genes at nucleotide codification (protein-coding and ribosomal genes). Maximum-likelihood tree was reconstructed using the best fit evolutionary model for the concatenated matrix. Numbers at nodes are statistical support values for bootstrap proportions. The scale bar is in expected substitutions/site. f Phylogenetic tree based on mt [file 13071_2024_6131_MOESM5_ESM.pdf]

Figure S1*a*.

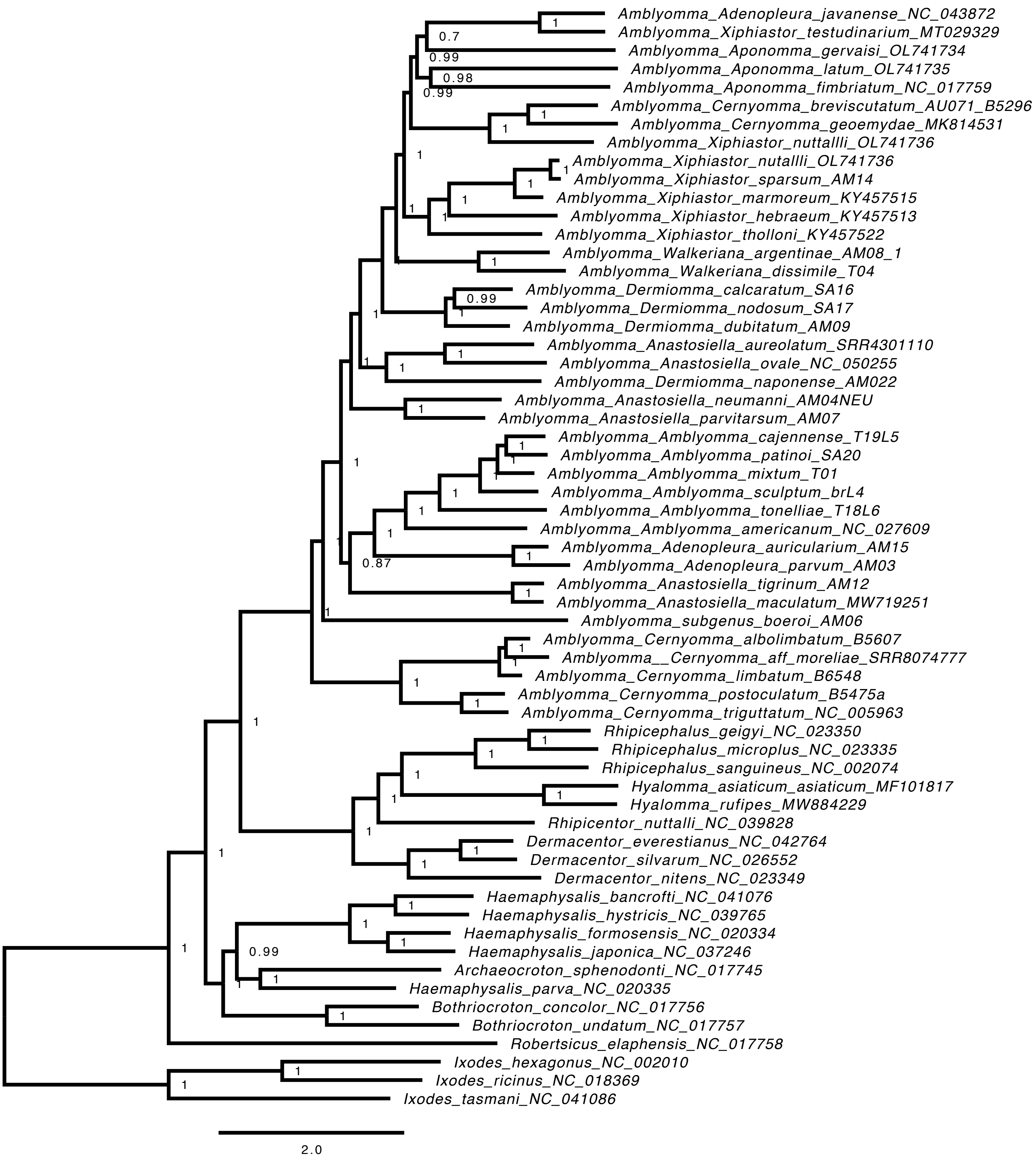
Amblyomma_Adenopleura_javanense_NC_043872
Amblyomma_Xiphiastor_testudinarium_MT029329
Amblyomma_Aponomma_gervaisi_OL741734
Amblyomma_Aponomma_latum_OL741735
Amblyomma_Aponomma_fimbriatum_NC_017759
Amblyomma_Cernyomma_breviscutatum_AU071_B5296
Amblyomma_Cernyomma_geoemydae_MK814531
Amblyomma_Xiphiastor_nuttallli_OL741736
Amblyomma_Xiphiastor_nutallli_OL741736
Amblyomma_Xiphiastor_sparsum_AM14
Amblyomma_Xiphiastor_marmoreum_KY457515
Amblyomma_Xiphiastor_hebraeum_KY457513
Amblyomma_Xiphiastor_tholloni_KY457522
Amblyomma_Walkeriana_argentinae_AM08_1
Amblyomma_Walkeriana_dissimile_T04
Amblyomma_Dermiomma_calcaratum_SA16
Amblyomma_Dermiomma_nodosum_SA17
Amblyomma_Dermiomma_dubitatum_AM09
Amblyomma_Anastosiella_aureolatum_SRR4301110
Amblyomma_Anastosiella_ovale_NC_050255
Amblyomma_Dermiomma_naponense_AM022
Amblyomma_Anastosiella_neumanni_AM04NEU
Amblyomma_Anastosiella_parvitarsum_AM07
Amblyomma_Amblyomma_cajennense_T19L5
Amblyomma_Amblyomma_patinoi_SA20
Amblyomma_Amblyomma_mixtum_T01
Amblyomma_Amblyomma_sculptum_brL4
Amblyomma_Amblyomma_tonelliae_T18L6
Amblyomma_Amblyomma_americanum_NC_027609
Amblyomma_Adenopleura_auricularium_AM15
Amblyomma_Adenopleura_parvum_AM03
Amblyomma_Anastosiella_tigrinum_AM12
Amblyomma_Anastosiella_maculatum_MW719251
Amblyomma_subgenus_boeroi_AM06
Amblyomma_Cernyomma_albolimbatum_B5607
Amblyomma__Cernyomma_aff_moreliae_SRR8074777
Amblyomma_Cernyomma_limbatum_B6548
Amblyomma_Cernyomma_postoculatum_B5475a
Amblyomma_Cernyomma_triguttatum_NC_005963
Rhipicephalus_geigyi_NC_023350
Rhipicephalus_microplus_NC_023335
Rhipicephalus_sanguineus_NC_002074
Hyalomma_asiaticum_asiaticum_MF101817
Hyalomma_rufipes_MW884229
Rhipicentor_nuttalli_NC_039828
Dermacentor_everestianus_NC_042764
Dermacentor_silvarum_NC_026552
Dermacentor_nitens_NC_023349
Haemaphysalis_bancrofti_NC_041076
Haemaphysalis_hystricis_NC_039765
Haemaphysalis_formosensis_NC_020334
Haemaphysalis_japonica_NC_037246
Archaeocroton_sphenodonti_NC_017745
Haemaphysalis_parva_NC_020335
Bothriocroton_concolor_NC_017756
Bothriocroton_undatum_NC_017757
Robertsicus_elaphensis_NC_017758
Ixodes_hexagonus_NC_002010
Ixodes_ricinus_NC_018369
Ixodes_tasmani_NC_041086
2.0

Figure S1*b*.

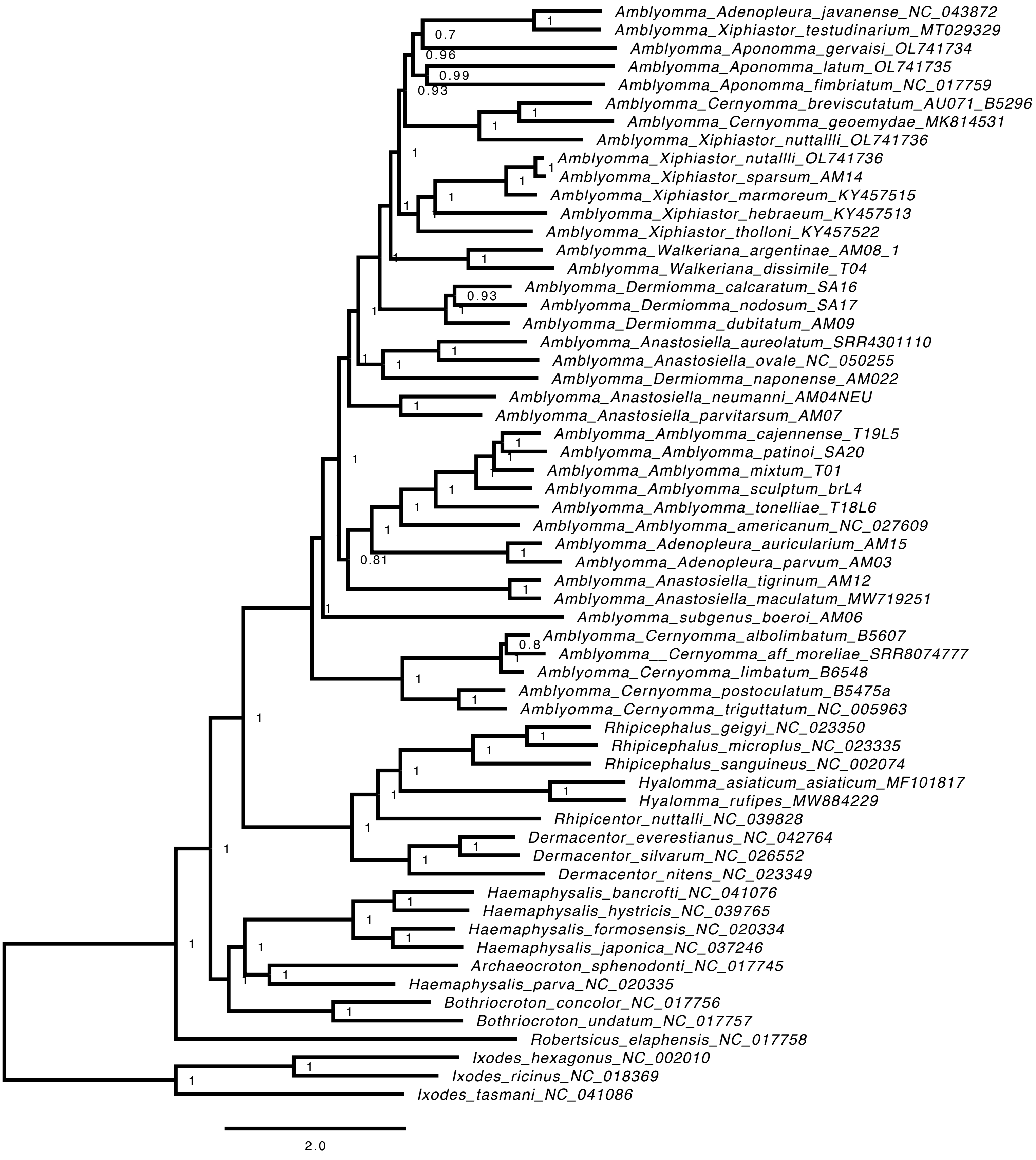
Amblyomma_Adenopleura_javanense_NC_043872
Amblyomma_Xiphiastor_testudinarium_MT029329
Amblyomma_Aponomma_gervaisi_OL741734
Amblyomma_Aponomma_latum_OL741735
Amblyomma_Aponomma_fimbriatum_NC_017759
Amblyomma_Cernyomma_breviscutatum_AU071_B5296
Amblyomma_Cernyomma_geoemydae_MK814531
Amblyomma_Xiphiastor_nuttallli_OL741736
Amblyomma_Xiphiastor_nutallli_OL741736
Amblyomma_Xiphiastor_sparsum_AM14
Amblyomma_Xiphiastor_marmoreum_KY457515
Amblyomma_Xiphiastor_hebraeum_KY457513
Amblyomma_Xiphiastor_tholloni_KY457522
Amblyomma_Walkeriana_argentinae_AM08_1
Amblyomma_Walkeriana_dissimile_T04
Amblyomma_Dermiomma_calcaratum_SA16
Amblyomma_Dermiomma_nodosum_SA17
Amblyomma_Dermiomma_dubitatum_AM09
Amblyomma_Anastosiella_aureolatum_SRR4301110
Amblyomma_Anastosiella_ovale_NC_050255
Amblyomma_Dermiomma_naponense_AM022
Amblyomma_Anastosiella_neumanni_AM04NEU
Amblyomma_Anastosiella_parvitarsum_AM07
Amblyomma_Amblyomma_cajennense_T19L5
Amblyomma_Amblyomma_patinoi_SA20
Amblyomma_Amblyomma_mixtum_T01
Amblyomma_Amblyomma_sculptum_brL4
Amblyomma_Amblyomma_tonelliae_T18L6
Amblyomma_Amblyomma_americanum_NC_027609
Amblyomma_Adenopleura_auricularium_AM15
Amblyomma_Adenopleura_parvum_AM03
Amblyomma_Anastosiella_tigrinum_AM12
Amblyomma_Anastosiella_maculatum_MW719251
Amblyomma_subgenus_boeroi_AM06
Amblyomma_Cernyomma_albolimbatum_B5607
Amblyomma__Cernyomma_aff_moreliae_SRR8074777
Amblyomma_Cernyomma_limbatum_B6548
Amblyomma_Cernyomma_postoculatum_B5475a
Amblyomma_Cernyomma_triguttatum_NC_005963
Rhipicephalus_geigyi_NC_023350
Rhipicephalus_microplus_NC_023335
Rhipicephalus_sanguineus_NC_002074
Hyalomma_asiaticum_asiaticum_MF101817
Hyalomma_rufipes_MW884229
Rhipicentor_nuttalli_NC_039828
Dermacentor_everestianus_NC_042764
Dermacentor_silvarum_NC_026552
Dermacentor_nitens_NC_023349
Haemaphysalis_bancrofti_NC_041076
Haemaphysalis_hystricis_NC_039765
Haemaphysalis_formosensis_NC_020334
Haemaphysalis_japonica_NC_037246
Archaeocroton_sphenodonti_NC_017745
Haemaphysalis_parva_NC_020335
Bothriocroton_concolor_NC_017756
Bothriocroton_undatum_NC_017757
Robertsicus_elaphensis_NC_017758
Ixodes_hexagonus_NC_002010
Ixodes_ricinus_NC_018369
Ixodes_tasmani_NC_041086
0.7
0.96
0.99
0.93
0.93
0.81
0.8
2.0

Figure S1*c*.

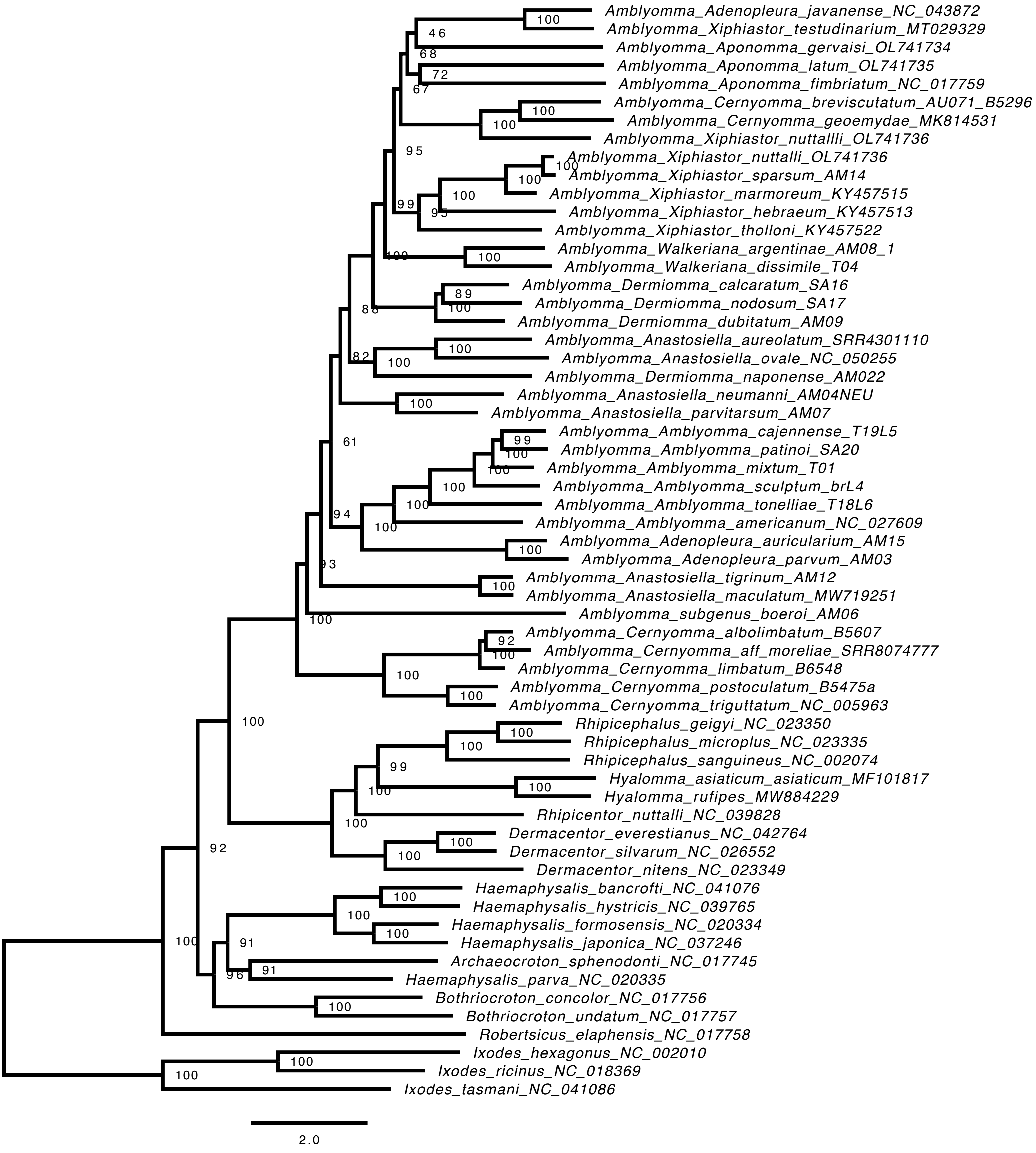
Amblyomma_Adenopleura_javanense_NC_043872
Amblyomma_Xiphiastor_testudinarium_MT029329
Amblyomma_Aponomma_gervaisi_OL741734
Amblyomma_Aponomma_latum_OL741735
Amblyomma_Aponomma_fimbriatum_NC_017759
Amblyomma_Cernyomma_breviscutatum_AU071_B5296
Amblyomma_Cernyomma_geoemydae_MK814531
Amblyomma_Xiphiastor_nuttallli_OL741736
Amblyomma_Xiphiastor_nuttalli_OL741736
Amblyomma_Xiphiastor_sparsum_AM14
Amblyomma_Xiphiastor_marmoreum_KY457515
Amblyomma_Xiphiastor_hebraeum_KY457513
Amblyomma_Xiphiastor_tholloni_KY457522
Amblyomma_Walkeriana_argentinae_AM08_1
Amblyomma_Walkeriana_dissimile_T04
Amblyomma_Dermiomma_calcaratum_SA16
Amblyomma_Dermiomma_nodosum_SA17
Amblyomma_Dermiomma_dubitatum_AM09
Amblyomma_Anastosiella_aureolatum_SRR4301110
Amblyomma_Anastosiella_ovale_NC_050255
Amblyomma_Dermiomma_naponense_AM022
Amblyomma_Anastosiella_neumanni_AM04NEU
Amblyomma_Anastosiella_parvitarsum_AM07
Amblyomma_Amblyomma_cajennense_T19L5
Amblyomma_Amblyomma_patinoi_SA20
Amblyomma_Amblyomma_mixtum_T01
Amblyomma_Amblyomma_sculptum_brL4
Amblyomma_Amblyomma_tonelliae_T18L6
Amblyomma_Amblyomma_americanum_NC_027609
Amblyomma_Adenopleura_auricularium_AM15
Amblyomma_Adenopleura_parvum_AM03
Amblyomma_Anastosiella_tigrinum_AM12
Amblyomma_Anastosiella_maculatum_MW719251
Amblyomma_subgenus_boeroi_AM06
Amblyomma_Cernyomma_albolimbatum_B5607
Amblyomma_Cernyomma_aff_moreliae_SRR8074777
Amblyomma_Cernyomma_limbatum_B6548
Amblyomma_Cernyomma_postoculatum_B5475a
Amblyomma_Cernyomma_triguttatum_NC_005963
Rhipicephalus_geigyi_NC_023350
Rhipicephalus_microplus_NC_023335
Rhipicephalus_sanguineus_NC_002074
Hyalomma_asiaticum_asiaticum_MF101817
Hyalomma_rufipes_MW884229
Rhipicentor_nuttalli_NC_039828
Dermacentor_everestianus_NC_042764
Dermacentor_silvarum_NC_026552
Dermacentor_nitens_NC_023349
Haemaphysalis_bancrofti_NC_041076
Haemaphysalis_hystricis_NC_039765
Haemaphysalis_formosensis_NC_020334
Haemaphysalis_japonica_NC_037246
Archaeocroton_sphenodonti_NC_017745
Haemaphysalis_parva_NC_020335
Bothriocroton_concolor_NC_017756
Bothriocroton_undatum_NC_017757
Robertsicus_elaphensis_NC_017758
Ixodes_hexagonus_NC_002010
Ixodes_ricinus_NC_018369
Ixodes_tasmani_NC_041086
2.0

Figure S1*d*.

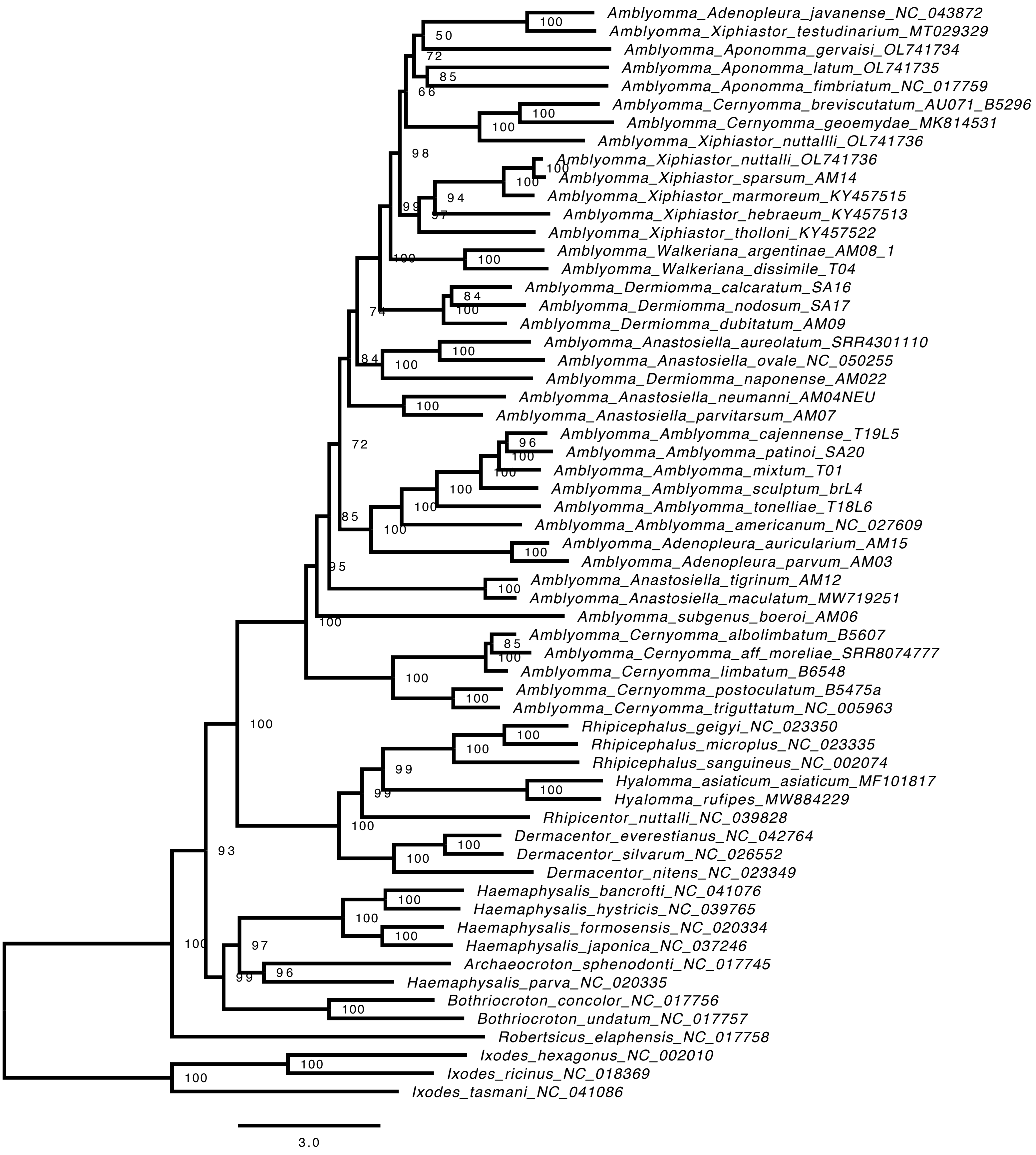

Amblyomma_Adenopleura_javanense_NC_043872
Amblyomma_Xiphiastor_testudinarium_MT029329
Amblyomma_Aponomma_gervaisi_OL741734
Amblyomma_Aponomma_latum_OL741735
Amblyomma_Aponomma_fimbriatum_NC_017759
Amblyomma_Cernyomma_breviscutatum_AU071_B5296
Amblyomma_Cernyomma_geoemydae_MK814531
Amblyomma_Xiphiastor_nuttallli_OL741736
Amblyomma_Xiphiastor_nuttalli_OL741736
Amblyomma_Xiphiastor_sparsum_AM14
Amblyomma_Xiphiastor_marmoreum_KY457515
Amblyomma_Xiphiastor_hebraeum_KY457513
Amblyomma_Xiphiastor_tholloni_KY457522
Amblyomma_Walkeriana_argentinae_AM08_1
Amblyomma_Walkeriana_dissimile_T04
Amblyomma_Dermiomma_calcaratum_SA16
Amblyomma_Dermiomma_nodosum_SA17
Amblyomma_Dermiomma_dubitatum_AM09
Amblyomma_Anastosiella_aureolatum_SRR4301110
Amblyomma_Anastosiella_ovale_NC_050255
Amblyomma_Dermiomma_naponense_AM022
Amblyomma_Anastosiella_neumanni_AM04NEU
Amblyomma_Anastosiella_parvitarsum_AM07
Amblyomma_Amblyomma_cajennense_T19L5
Amblyomma_Amblyomma_patinoi_SA20
Amblyomma_Amblyomma_mixtum_T01
Amblyomma_Amblyomma_sculptum_brL4
Amblyomma_Amblyomma_tonelliae_T18L6
Amblyomma_Amblyomma_americanum_NC_027609
Amblyomma_Adenopleura_auricularium_AM15
Amblyomma_Adenopleura_parvum_AM03
Amblyomma_Anastosiella_tigrinum_AM12
Amblyomma_Anastosiella_maculatum_MW719251
Amblyomma_subgenus_boeroi_AM06
Amblyomma_Cernyomma_albolimbatum_B5607
Amblyomma_Cernyomma_aff_moreliae_SRR8074777
Amblyomma_Cernyomma_limbatum_B6548
Amblyomma_Cernyomma_postoculatum_B5475a
Amblyomma_Cernyomma_triguttatum_NC_005963
Rhipicephalus_geigyi_NC_023350
Rhipicephalus_microplus_NC_023335
Rhipicephalus_sanguineus_NC_002074
Hyalomma_asiaticum_asiaticum_MF101817
Hyalomma_rufipes_MW884229
Rhipicentor_nuttalli_NC_039828
Dermacentor_everestianus_NC_042764
Dermacentor_silvarum_NC_026552
Dermacentor_nitens_NC_023349
Haemaphysalis_bancrofti_NC_041076
Haemaphysalis_hystricis_NC_039765
Haemaphysalis_formosensis_NC_020334
Haemaphysalis_japonica_NC_037246
Archaeocroton_sphenodonti_NC_017745
Haemaphysalis_parva_NC_020335
Bothriocroton_concolor_NC_017756
Bothriocroton_undatum_NC_017757
Robertsicus_elaphensis_NC_017758
Ixodes_hexagonus_NC_002010
Ixodes_ricinus_NC_018369
Ixodes_tasmani_NC_041086
100
50
72
85
66
100
100
98
100
100
94
99
97
100
100
84
100
74
100
84
100
100
72
96
100
100
100
100
85
100
100
95
100
100
85
100
100
100
100
100
100
99
99
100
100
100
100
93
100
100
100
100
97
99
96
100
100
100
3.0

Figure S1*e.*

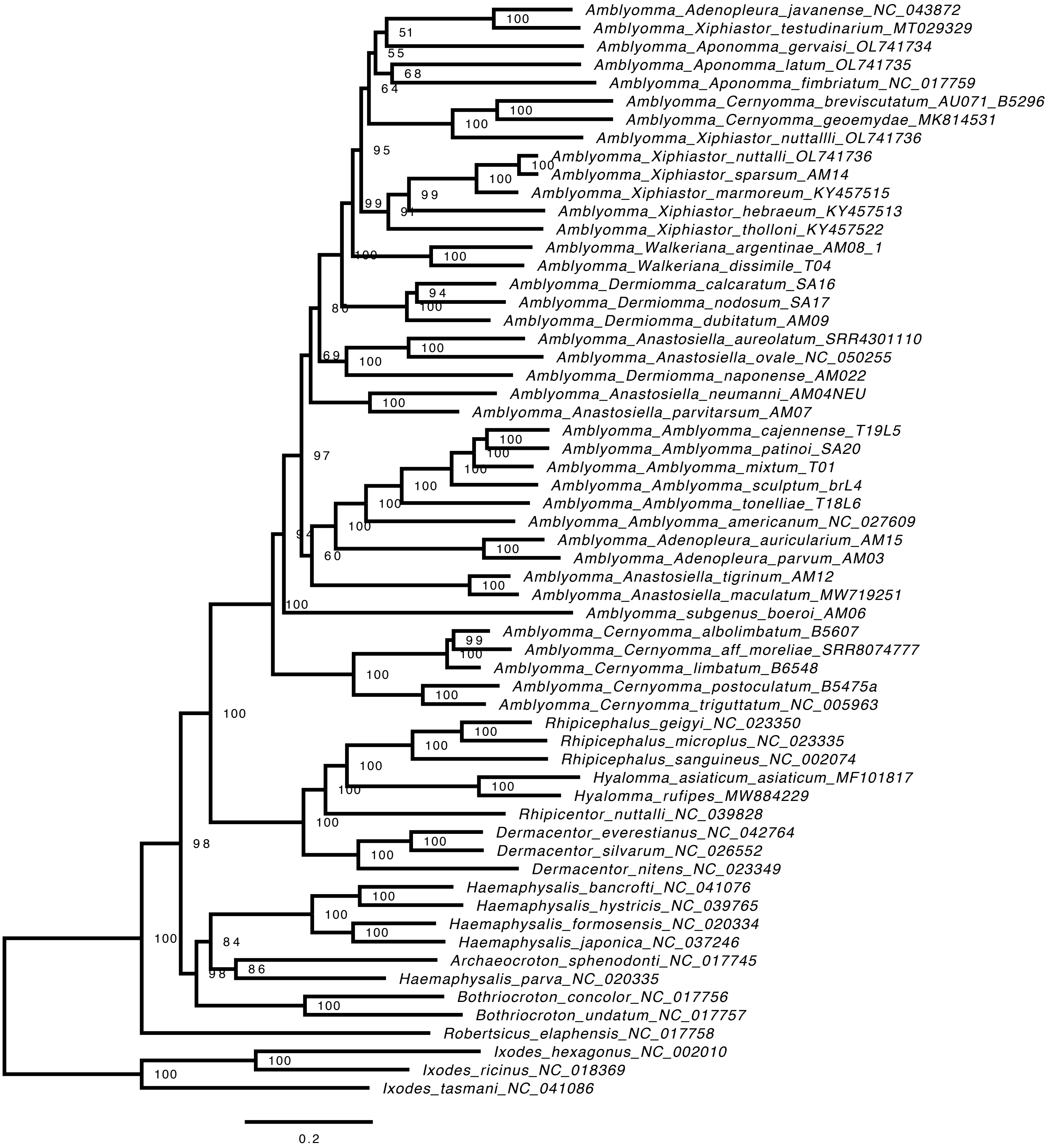

Amblyomma_Adenopleura_javanense_NC_043872
Amblyomma_Xiphiastor_testudinarium_MT029329
Amblyomma_Aponomma_gervaisi_OL741734
Amblyomma_Aponomma_latum_OL741735
Amblyomma_Aponomma_fimbriatum_NC_017759
Amblyomma_Cernyomma_breviscutatum_AU071_B5296
Amblyomma_Cernyomma_geoemydae_MK814531
Amblyomma_Xiphiastor_nuttallli_OL741736
Amblyomma_Xiphiastor_nuttalli_OL741736
Amblyomma_Xiphiastor_sparsum_AM14
Amblyomma_Xiphiastor_marmoreum_KY457515
Amblyomma_Xiphiastor_hebraeum_KY457513
Amblyomma_Xiphiastor_tholloni_KY457522
Amblyomma_Walkeriana_argentinae_AM08_1
Amblyomma_Walkeriana_dissimile_T04
Amblyomma_Dermiomma_calcaratum_SA16
Amblyomma_Dermiomma_nodosum_SA17
Amblyomma_Dermiomma_dubitatum_AM09
Amblyomma_Anastosiella_aureolatum_SRR4301110
Amblyomma_Anastosiella_ovale_NC_050255
Amblyomma_Dermiomma_naponense_AM022
Amblyomma_Anastosiella_neumanni_AM04NEU
Amblyomma_Anastosiella_parvitarsum_AM07
Amblyomma_Amblyomma_cajennense_T19L5
Amblyomma_Amblyomma_patinoi_SA20
Amblyomma_Amblyomma_mixtum_T01
Amblyomma_Amblyomma_sculptum_brL4
Amblyomma_Amblyomma_tonelliae_T18L6
Amblyomma_Amblyomma_americanum_NC_027609
Amblyomma_Adenopleura_auricularium_AM15
Amblyomma_Adenopleura_parvum_AM03
Amblyomma_Anastosiella_tigrinum_AM12
Amblyomma_Anastosiella_maculatum_MW719251
Amblyomma_subgenus_boeroi_AM06
Amblyomma_Cernyomma_albolimbatum_B5607
Amblyomma_Cernyomma_aff_moreliae_SRR8074777
Amblyomma_Cernyomma_limbatum_B6548
Amblyomma_Cernyomma_postoculatum_B5475a
Amblyomma_Cernyomma_triguttatum_NC_005963
Rhipicephalus_geigyi_NC_023350
Rhipicephalus_microplus_NC_023335
Rhipicephalus_sanguineus_NC_002074
Hyalomma_asiaticum_asiaticum_MF101817
Hyalomma_rufipes_MW884229
Rhipicentor_nuttalli_NC_039828
Dermacentor_everestianus_NC_042764
Dermacentor_silvarum_NC_026552
Dermacentor_nitens_NC_023349
Haemaphysalis_bancrofti_NC_041076
Haemaphysalis_hystricis_NC_039765
Haemaphysalis_formosensis_NC_020334
Haemaphysalis_japonica_NC_037246
Archaeocroton_sphenodonti_NC_017745
Haemaphysalis_parva_NC_020335
Bothriocroton_concolor_NC_017756
Bothriocroton_undatum_NC_017757
Robertsicus_elaphensis_NC_017758
Ixodes_hexagonus_NC_002010
Ixodes_ricinus_NC_018369
Ixodes_tasmani_NC_041086
0.2

Figure S1*f*.

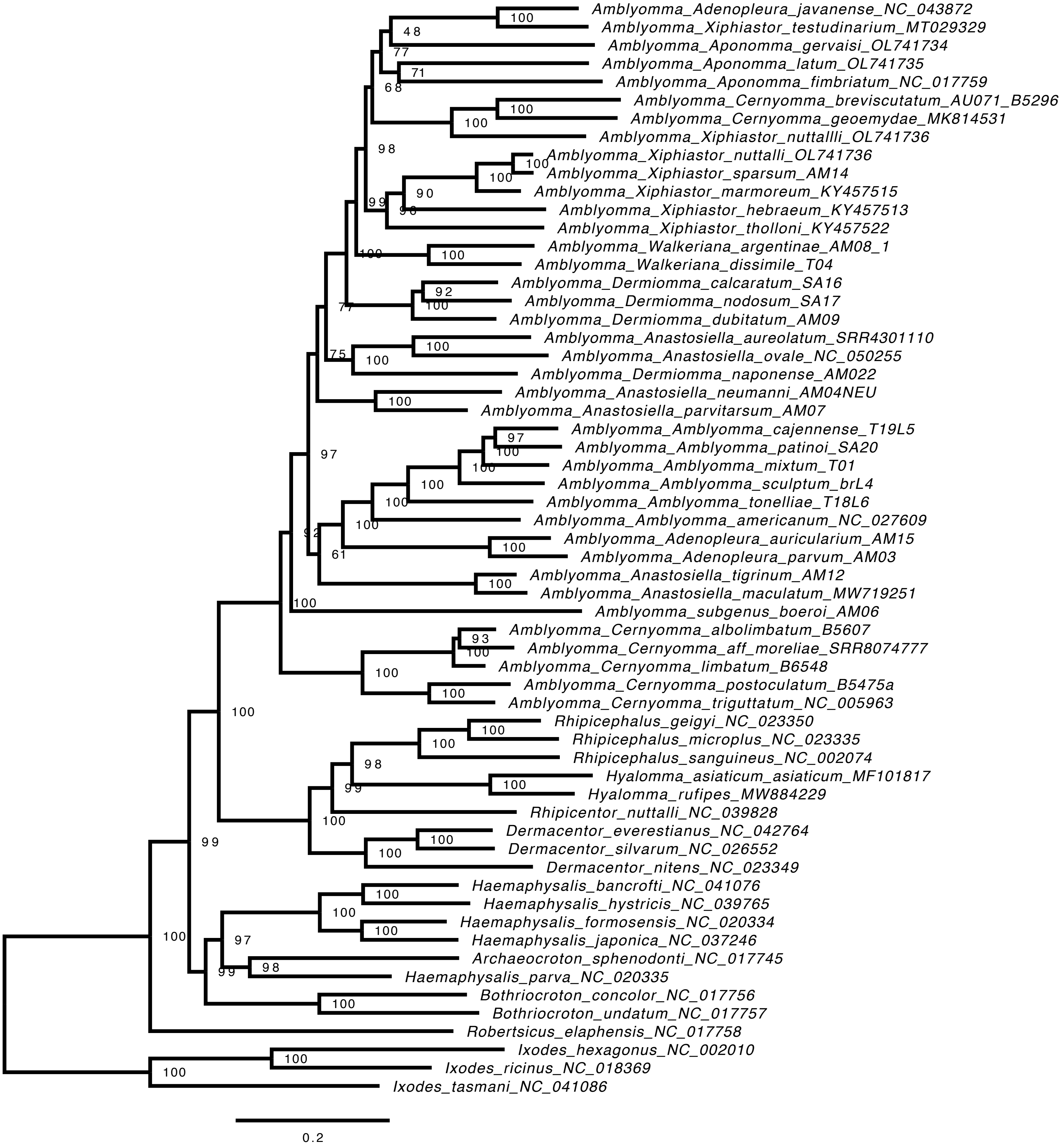
Amblyomma_Adenopleura_javanense_NC_043872
Amblyomma_Xiphiastor_testudinarium_MT029329
Amblyomma_Aponomma_gervaisi_OL741734
Amblyomma_Aponomma_latum_OL741735
Amblyomma_Aponomma_fimbriatum_NC_017759
Amblyomma_Cernyomma_breviscutatum_AU071_B5296
Amblyomma_Cernyomma_geoemydae_MK814531
Amblyomma_Xiphiastor_nuttallli_OL741736
Amblyomma_Xiphiastor_nuttalli_OL741736
Amblyomma_Xiphiastor_sparsum_AM14
Amblyomma_Xiphiastor_marmoreum_KY457515
Amblyomma_Xiphiastor_hebraeum_KY457513
Amblyomma_Xiphiastor_tholloni_KY457522
Amblyomma_Walkeriana_argentinae_AM08_1
Amblyomma_Walkeriana_dissimile_T04
Amblyomma_Dermiomma_calcaratum_SA16
Amblyomma_Dermiomma_nodosum_SA17
Amblyomma_Dermiomma_dubitatum_AM09
Amblyomma_Anastosiella_aureolatum_SRR4301110
Amblyomma_Anastosiella_ovale_NC_050255
Amblyomma_Dermiomma_naponense_AM022
Amblyomma_Anastosiella_neumanni_AM04NEU
Amblyomma_Anastosiella_parvitarsum_AM07
Amblyomma_Amblyomma_cajennense_T19L5
Amblyomma_Amblyomma_patinoi_SA20
Amblyomma_Amblyomma_mixtum_T01
Amblyomma_Amblyomma_sculptum_brL4
Amblyomma_Amblyomma_tonelliae_T18L6
Amblyomma_Amblyomma_americanum_NC_027609
Amblyomma_Adenopleura_auricularium_AM15
Amblyomma_Adenopleura_parvum_AM03
Amblyomma_Anastosiella_tigrinum_AM12
Amblyomma_Anastosiella_maculatum_MW719251
Amblyomma_subgenus_boeroi_AM06
Amblyomma_Cernyomma_albolimbatum_B5607
Amblyomma_Cernyomma_aff_moreliae_SRR8074777
Amblyomma_Cernyomma_limbatum_B6548
Amblyomma_Cernyomma_postoculatum_B5475a
Amblyomma_Cernyomma_triguttatum_NC_005963
Rhipicephalus_geigyi_NC_023350
Rhipicephalus_microplus_NC_023335
Rhipicephalus_sanguineus_NC_002074
Hyalomma_asiaticum_asiaticum_MF101817
Hyalomma_rufipes_MW884229
Rhipicentor_nuttalli_NC_039828
Dermacentor_everestianus_NC_042764
Dermacentor_silvarum_NC_026552
Dermacentor_nitens_NC_023349
Haemaphysalis_bancrofti_NC_041076
Haemaphysalis_hystricis_NC_039765
Haemaphysalis_formosensis_NC_020334
Haemaphysalis_japonica_NC_037246
Archaeocroton_sphenodonti_NC_017745
Haemaphysalis_parva_NC_020335
Bothriocroton_concolor_NC_017756
Bothriocroton_undatum_NC_017757
Robertsicus_elaphensis_NC_017758
Ixodes_hexagonus_NC_002010
Ixodes_ricinus_NC_018369
Ixodes_tasmani_NC_041086
0.2

Figure S1g.

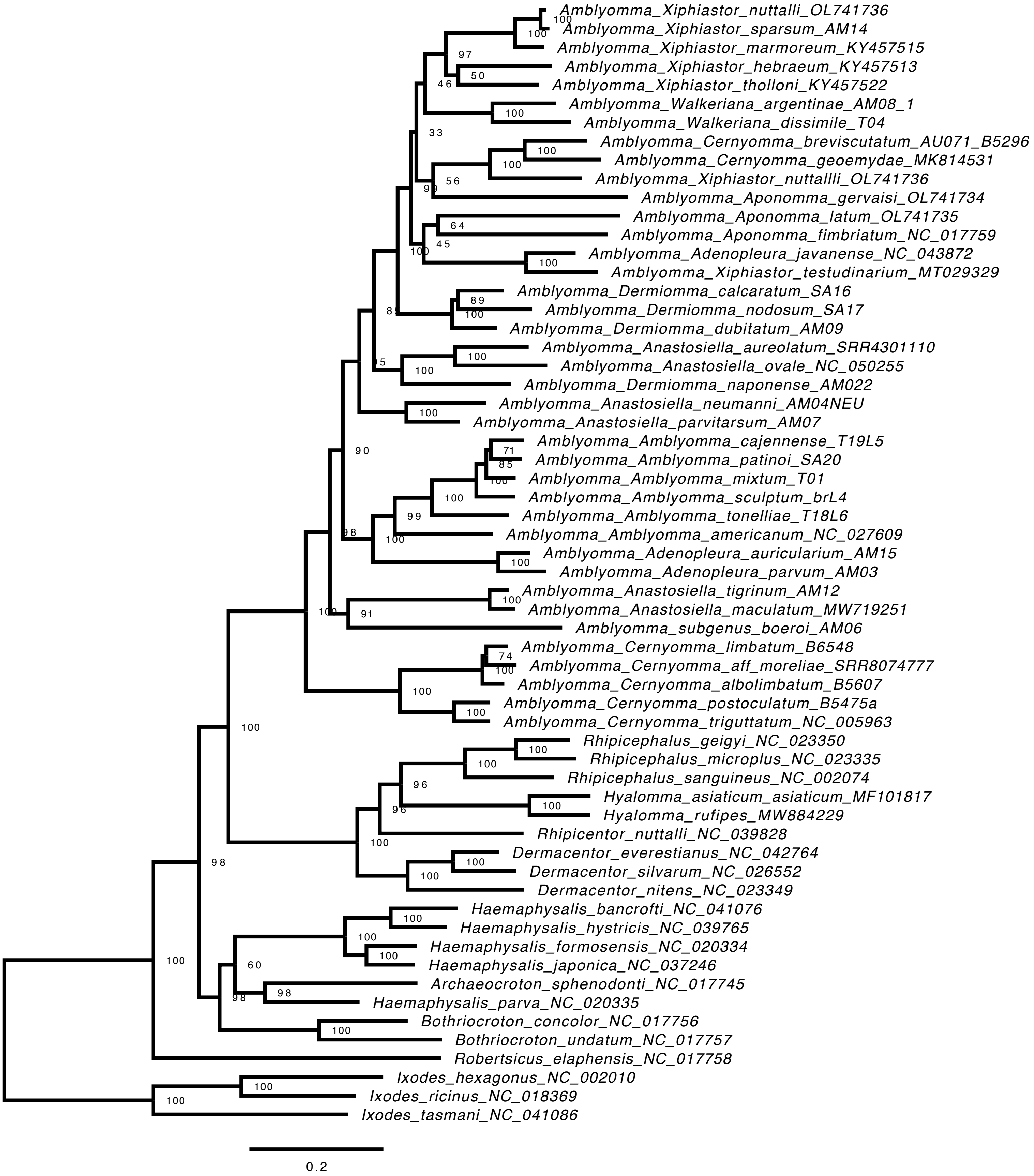

Amblyomma_Xiphiastor_nuttalli_OL741736
Amblyomma_Xiphiastor_sparsum_AM14
Amblyomma_Xiphiastor_marmoreum_KY457515
Amblyomma_Xiphiastor_hebraeum_KY457513
Amblyomma_Xiphiastor_tholloni_KY457522
Amblyomma_Walkeriana_argentinae_AM08_1
Amblyomma_Walkeriana_dissimile_T04
Amblyomma_Cernyomma_breviscutatum_AU071_B5296
Amblyomma_Cernyomma_geoemydae_MK814531
Amblyomma_Xiphiastor_nuttallli_OL741736
Amblyomma_Aponomma_gervaisi_OL741734
Amblyomma_Aponomma_latum_OL741735
Amblyomma_Aponomma_fimbriatum_NC_017759
Amblyomma_Adenopleura_javanense_NC_043872
Amblyomma_Xiphiastor_testudinarium_MT029329
Amblyomma_Dermiomma_calcaratum_SA16
Amblyomma_Dermiomma_nodosum_SA17
Amblyomma_Dermiomma_dubitatum_AM09
Amblyomma_Anastosiella_aureolatum_SRR4301110
Amblyomma_Anastosiella_ovale_NC_050255
Amblyomma_Dermiomma_naponense_AM022
Amblyomma_Anastosiella_neumanni_AM04NEU
Amblyomma_Anastosiella_parvitarsum_AM07
Amblyomma_Amblyomma_cajennense_T19L5
Amblyomma_Amblyomma_patinoi_SA20
Amblyomma_Amblyomma_mixtum_T01
Amblyomma_Amblyomma_sculptum_brL4
Amblyomma_Amblyomma_tonelliae_T18L6
Amblyomma_Amblyomma_americanum_NC_027609
Amblyomma_Adenopleura_auricularium_AM15
Amblyomma_Adenopleura_parvum_AM03
Amblyomma_Anastosiella_tigrinum_AM12
Amblyomma_Anastosiella_maculatum_MW719251
Amblyomma_subgenus_boeroi_AM06
Amblyomma_Cernyomma_limbatum_B6548
Amblyomma_Cernyomma_aff_moreliae_SRR8074777
Amblyomma_Cernyomma_albolimbatum_B5607
Amblyomma_Cernyomma_postoculatum_B5475a
Amblyomma_Cernyomma_triguttatum_NC_005963
Rhipicephalus_geigyi_NC_023350
Rhipicephalus_microplus_NC_023335
Rhipicephalus_sanguineus_NC_002074
Hyalomma_asiaticum_asiaticum_MF101817
Hyalomma_rufipes_MW884229
Rhipicentor_nuttalli_NC_039828
Dermacentor_everestianus_NC_042764
Dermacentor_silvarum_NC_026552
Dermacentor_nitens_NC_023349
Haemaphysalis_bancrofti_NC_041076
Haemaphysalis_hystricis_NC_039765
Haemaphysalis_formosensis_NC_020334
Haemaphysalis_japonica_NC_037246
Archaeocroton_sphenodonti_NC_017745
Haemaphysalis_parva_NC_020335
Bothriocroton_concolor_NC_017756
Bothriocroton_undatum_NC_017757
Robertsicus_elaphensis_NC_017758
Ixodes_hexagonus_NC_002010
Ixodes_ricinus_NC_018369
Ixodes_tasmani_NC_041086
0.2

Figure S1*h*.

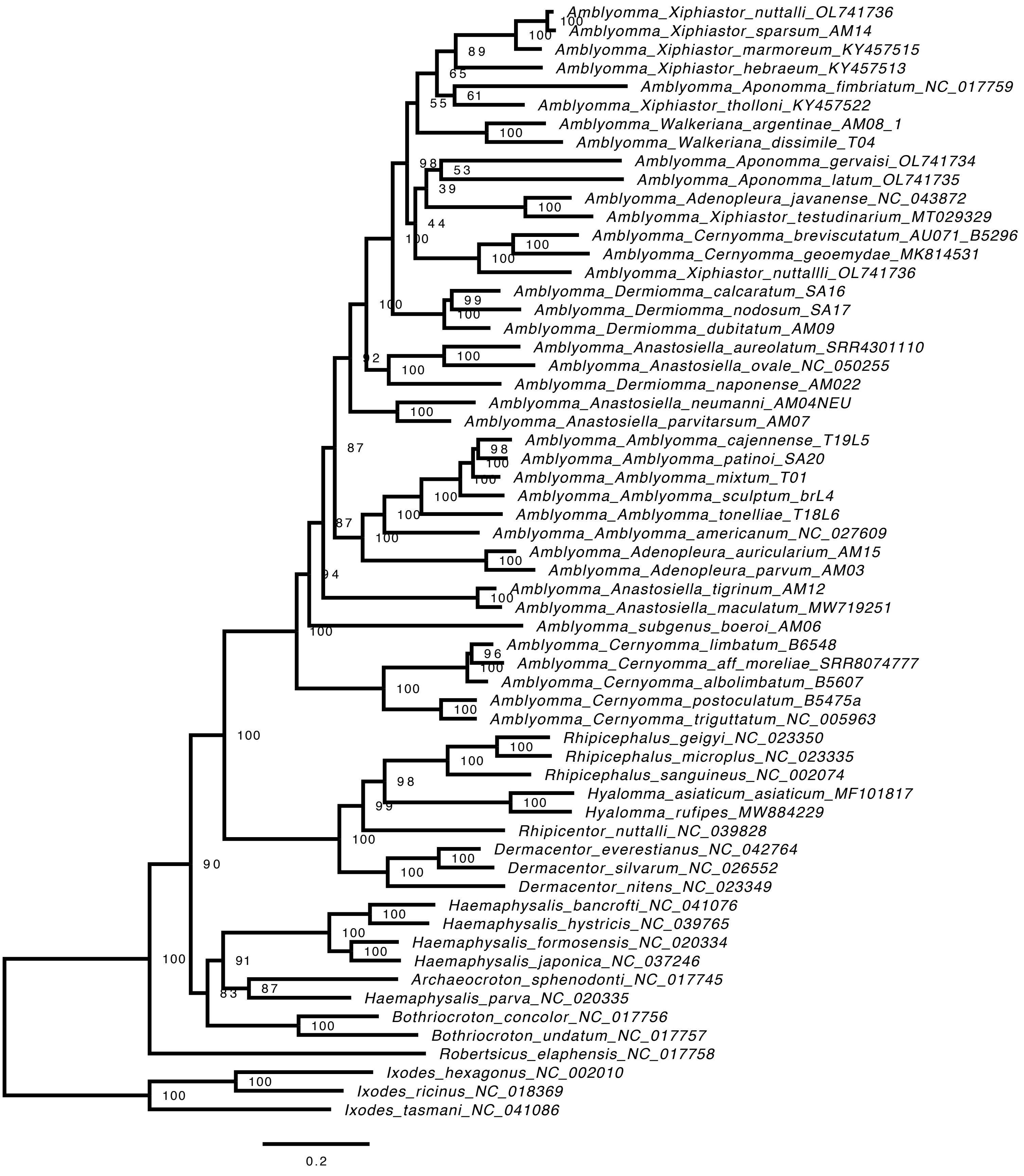
Amblyomma_Xiphiastor_nuttalli_OL741736
Amblyomma_Xiphiastor_sparsum_AM14
Amblyomma_Xiphiastor_marmoreum_KY457515
Amblyomma_Xiphiastor_hebraeum_KY457513
Amblyomma_Aponomma_fimbriatum_NC_017759
Amblyomma_Xiphiastor_tholloni_KY457522
Amblyomma_Walkeriana_argentinae_AM08_1
Amblyomma_Walkeriana_dissimile_T04
Amblyomma_Aponomma_gervaisi_OL741734
Amblyomma_Aponomma_latum_OL741735
Amblyomma_Adenopleura_javanense_NC_043872
Amblyomma_Xiphiastor_testudinarium_MT029329
Amblyomma_Cernyomma_breviscutatum_AU071_B5296
Amblyomma_Cernyomma_geoemydae_MK814531
Amblyomma_Xiphiastor_nuttallli_OL741736
Amblyomma_Dermiomma_calcaratum_SA16
Amblyomma_Dermiomma_nodosum_SA17
Amblyomma_Dermiomma_dubitatum_AM09
Amblyomma_Anastosiella_aureolatum_SRR4301110
Amblyomma_Anastosiella_ovale_NC_050255
Amblyomma_Dermiomma_naponense_AM022
Amblyomma_Anastosiella_neumanni_AM04NEU
Amblyomma_Anastosiella_parvitarsum_AM07
Amblyomma_Amblyomma_cajennense_T19L5
Amblyomma_Amblyomma_patinoi_SA20
Amblyomma_Amblyomma_mixtum_T01
Amblyomma_Amblyomma_sculptum_brL4
Amblyomma_Amblyomma_tonelliae_T18L6
Amblyomma_Amblyomma_americanum_NC_027609
Amblyomma_Adenopleura_auricularium_AM15
Amblyomma_Adenopleura_parvum_AM03
Amblyomma_Anastosiella_tigrinum_AM12
Amblyomma_Anastosiella_maculatum_MW719251
Amblyomma_subgenus_boeroi_AM06
Amblyomma_Cernyomma_limbatum_B6548
Amblyomma_Cernyomma_aff_moreliae_SRR8074777
Amblyomma_Cernyomma_albolimbatum_B5607
Amblyomma_Cernyomma_postoculatum_B5475a
Amblyomma_Cernyomma_triguttatum_NC_005963
Rhipicephalus_geigyi_NC_023350
Rhipicephalus_microplus_NC_023335
Rhipicephalus_sanguineus_NC_002074
Hyalomma_asiaticum_asiaticum_MF101817
Hyalomma_rufipes_MW884229
Rhipicentor_nuttalli_NC_039828
Dermacentor_everestianus_NC_042764
Dermacentor_silvarum_NC_026552
Dermacentor_nitens_NC_023349
Haemaphysalis_bancrofti_NC_041076
Haemaphysalis_hystricis_NC_039765
Haemaphysalis_formosensis_NC_020334
Haemaphysalis_japonica_NC_037246
Archaeocroton_sphenodonti_NC_017745
Haemaphysalis_parva_NC_020335
Bothriocroton_concolor_NC_017756
Bothriocroton_undatum_NC_017757
Robertsicus_elaphensis_NC_017758
Ixodes_hexagonus_NC_002010
Ixodes_ricinus_NC_018369
Ixodes_tasmani_NC_041086
100
100
89
65
61
55
100
98
53
39
100
44
100
100
100
99
100
100
92
100
100
87
98
100
100
100
100
87
100
100
94
100
100
96
100
100
100
100
100
100
98
99
100
100
100
100
90
100
100
100
91
87
83
100
100
100
0.2

Figure S1*i*.

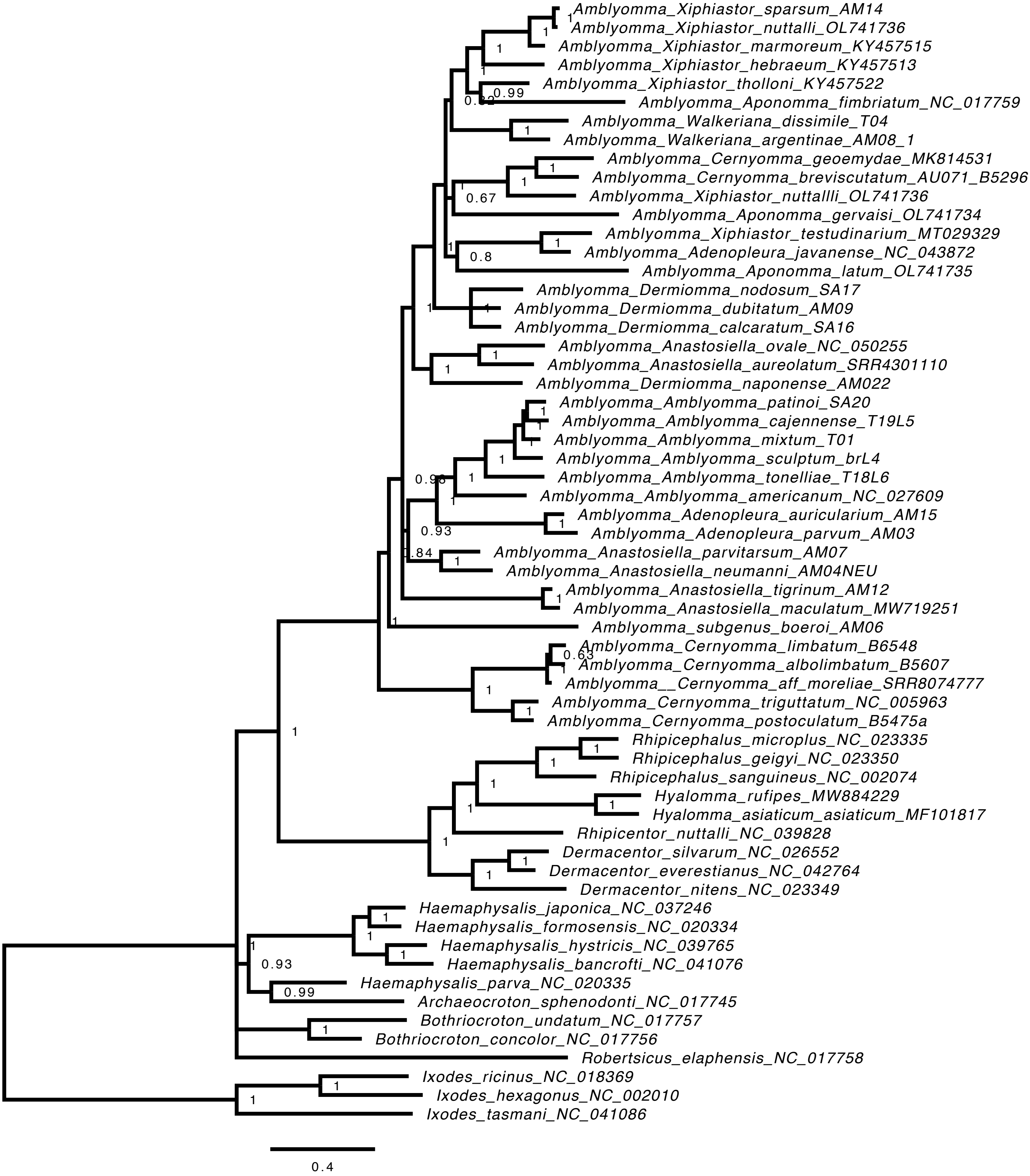

Amblyomma_Xiphiastor_sparsum_AM14
Amblyomma_Xiphiastor_nuttalli_OL741736
Amblyomma_Xiphiastor_marmoreum_KY457515
Amblyomma_Xiphiastor_hebraeum_KY457513
Amblyomma_Xiphiastor_tholloni_KY457522
Amblyomma_Aponomma_fimbriatum_NC_017759
Amblyomma_Walkeriana_dissimile_T04
Amblyomma_Walkeriana_argentinae_AM08_1
Amblyomma_Cernyomma_geoemydae_MK814531
Amblyomma_Cernyomma_breviscutatum_AU071_B5296
Amblyomma_Xiphiastor_nuttallli_OL741736
Amblyomma_Aponomma_gervaisi_OL741734
Amblyomma_Xiphiastor_testudinarium_MT029329
Amblyomma_Adenopleura_javanense_NC_043872
Amblyomma_Aponomma_latum_OL741735
Amblyomma_Dermiomma_nodosum_SA17
Amblyomma_Dermiomma_dubitatum_AM09
Amblyomma_Dermiomma_calcaratum_SA16
Amblyomma_Anastosiella_ovale_NC_050255
Amblyomma_Anastosiella_aureolatum_SRR4301110
Amblyomma_Dermiomma_naponense_AM022
Amblyomma_Amblyomma_patinoi_SA20
Amblyomma_Amblyomma_cajennense_T19L5
Amblyomma_Amblyomma_mixtum_T01
Amblyomma_Amblyomma_sculptum_brL4
Amblyomma_Amblyomma_tonelliae_T18L6
Amblyomma_Amblyomma_americanum_NC_027609
Amblyomma_Adenopleura_auricularium_AM15
Amblyomma_Adenopleura_parvum_AM03
Amblyomma_Anastosiella_parvitarsum_AM07
Amblyomma_Anastosiella_neumanni_AM04NEU
Amblyomma_Anastosiella_tigrinum_AM12
Amblyomma_Anastosiella_maculatum_MW719251
Amblyomma_subgenus_boeroi_AM06
Amblyomma_Cernyomma_limbatum_B6548
Amblyomma_Cernyomma_albolimbatum_B5607
Amblyomma__Cernyomma_aff_moreliae_SRR8074777
Amblyomma_Cernyomma_triguttatum_NC_005963
Amblyomma_Cernyomma_postoculatum_B5475a
Rhipicephalus_microplus_NC_023335
Rhipicephalus_geigyi_NC_023350
Rhipicephalus_sanguineus_NC_002074
Hyalomma_rufipes_MW884229
Hyalomma_asiaticum_asiaticum_MF101817
Rhipicentor_nuttalli_NC_039828
Dermacentor_silvarum_NC_026552
Dermacentor_everestianus_NC_042764
Dermacentor_nitens_NC_023349
Haemaphysalis_japonica_NC_037246
Haemaphysalis_formosensis_NC_020334
Haemaphysalis_hystricis_NC_039765
Haemaphysalis_bancrofti_NC_041076
Haemaphysalis_parva_NC_020335
Archaeocroton_sphenodonti_NC_017745
Bothriocroton_undatum_NC_017757
Bothriocroton_concolor_NC_017756
Robertsicus_elaphensis_NC_017758
Ixodes_ricinus_NC_018369
Ixodes_hexagonus_NC_002010
Ixodes_tasmani_NC_041086
0.99
0.67
0.8
0.93
0.63
0.99
0.93
0.4

Figure S1*j*.

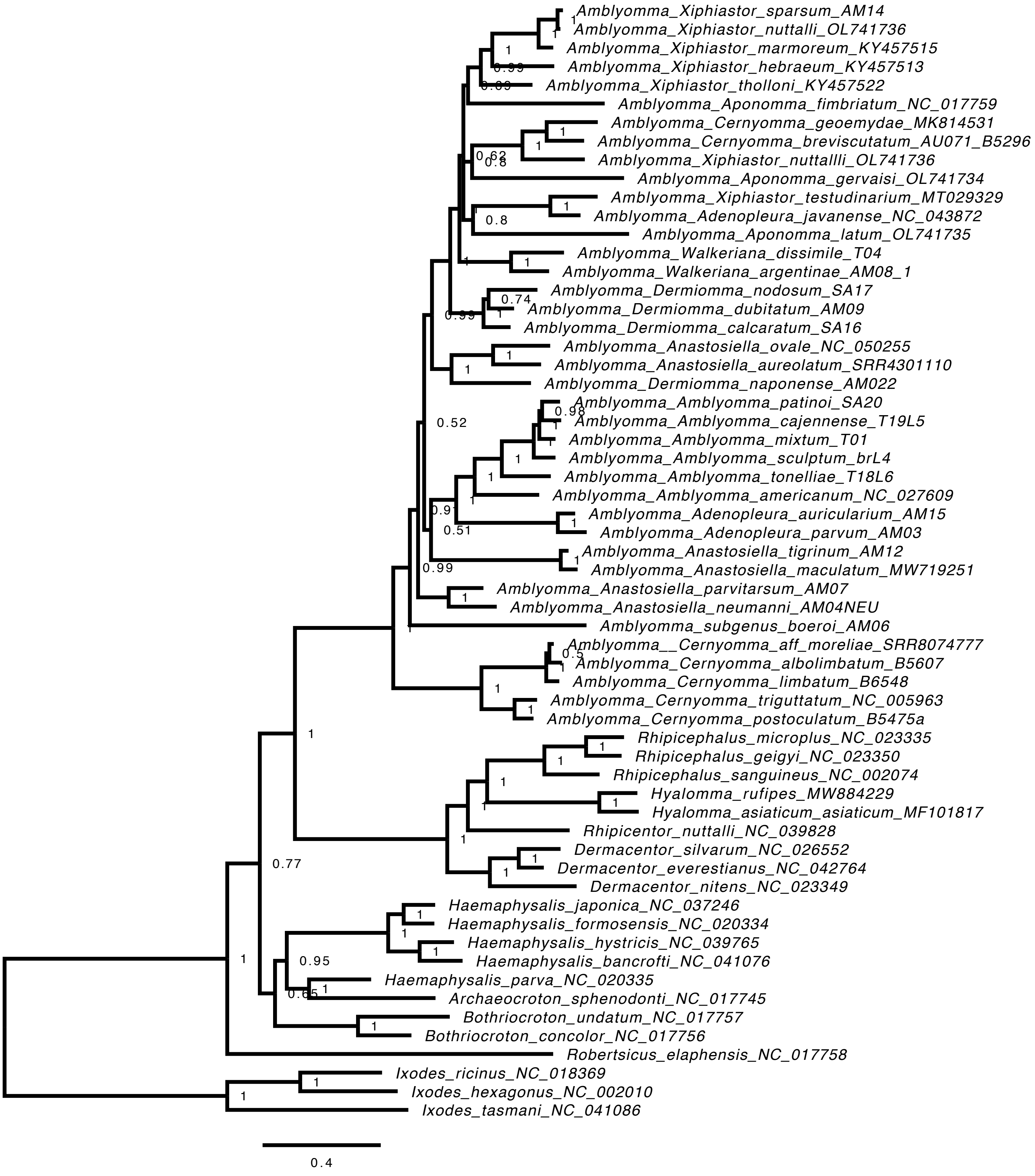
Amblyomma_Xiphiastor_sparsum_AM14
Amblyomma_Xiphiastor_nuttalli_OL741736
Amblyomma_Xiphiastor_marmoreum_KY457515
Amblyomma_Xiphiastor_hebraeum_KY457513
Amblyomma_Xiphiastor_tholloni_KY457522
Amblyomma_Aponomma_fimbriatum_NC_017759
Amblyomma_Cernyomma_geoemydae_MK814531
Amblyomma_Cernyomma_breviscutatum_AU071_B5296
Amblyomma_Xiphiastor_nuttallli_OL741736
Amblyomma_Aponomma_gervaisi_OL741734
Amblyomma_Xiphiastor_testudinarium_MT029329
Amblyomma_Adenopleura_javanense_NC_043872
Amblyomma_Aponomma_latum_OL741735
Amblyomma_Walkeriana_dissimile_T04
Amblyomma_Walkeriana_argentinae_AM08_1
Amblyomma_Dermiomma_nodosum_SA17
Amblyomma_Dermiomma_dubitatum_AM09
Amblyomma_Dermiomma_calcaratum_SA16
Amblyomma_Anastosiella_ovale_NC_050255
Amblyomma_Anastosiella_aureolatum_SRR4301110
Amblyomma_Dermiomma_naponense_AM022
Amblyomma_Amblyomma_patinoi_SA20
Amblyomma_Amblyomma_cajennense_T19L5
Amblyomma_Amblyomma_mixtum_T01
Amblyomma_Amblyomma_sculptum_brL4
Amblyomma_Amblyomma_tonelliae_T18L6
Amblyomma_Amblyomma_americanum_NC_027609
Amblyomma_Adenopleura_auricularium_AM15
Amblyomma_Adenopleura_parvum_AM03
Amblyomma_Anastosiella_tigrinum_AM12
Amblyomma_Anastosiella_maculatum_MW719251
Amblyomma_Anastosiella_parvitarsum_AM07
Amblyomma_Anastosiella_neumanni_AM04NEU
Amblyomma_subgenus_boeroi_AM06
Amblyomma__Cernyomma_aff_moreliae_SRR8074777
Amblyomma_Cernyomma_albolimbatum_B5607
Amblyomma_Cernyomma_limbatum_B6548
Amblyomma_Cernyomma_triguttatum_NC_005963
Amblyomma_Cernyomma_postoculatum_B5475a
Rhipicephalus_microplus_NC_023335
Rhipicephalus_geigyi_NC_023350
Rhipicephalus_sanguineus_NC_002074
Hyalomma_rufipes_MW884229
Hyalomma_asiaticum_asiaticum_MF101817
Rhipicentor_nuttalli_NC_039828
Dermacentor_silvarum_NC_026552
Dermacentor_everestianus_NC_042764
Dermacentor_nitens_NC_023349
Haemaphysalis_japonica_NC_037246
Haemaphysalis_formosensis_NC_020334
Haemaphysalis_hystricis_NC_039765
Haemaphysalis_bancrofti_NC_041076
Haemaphysalis_parva_NC_020335
Archaeocroton_sphenodonti_NC_017745
Bothriocroton_undatum_NC_017757
Bothriocroton_concolor_NC_017756
Robertsicus_elaphensis_NC_017758
Ixodes_ricinus_NC_018369
Ixodes_hexagonus_NC_002010
Ixodes_tasmani_NC_041086
0.99
0.99
0.62
0.8
0.8
0.74
0.99
0.52
0.98
0.91
0.51
0.99
0.5
0.77
0.95
0.65
0.4

Figure S1*k*.

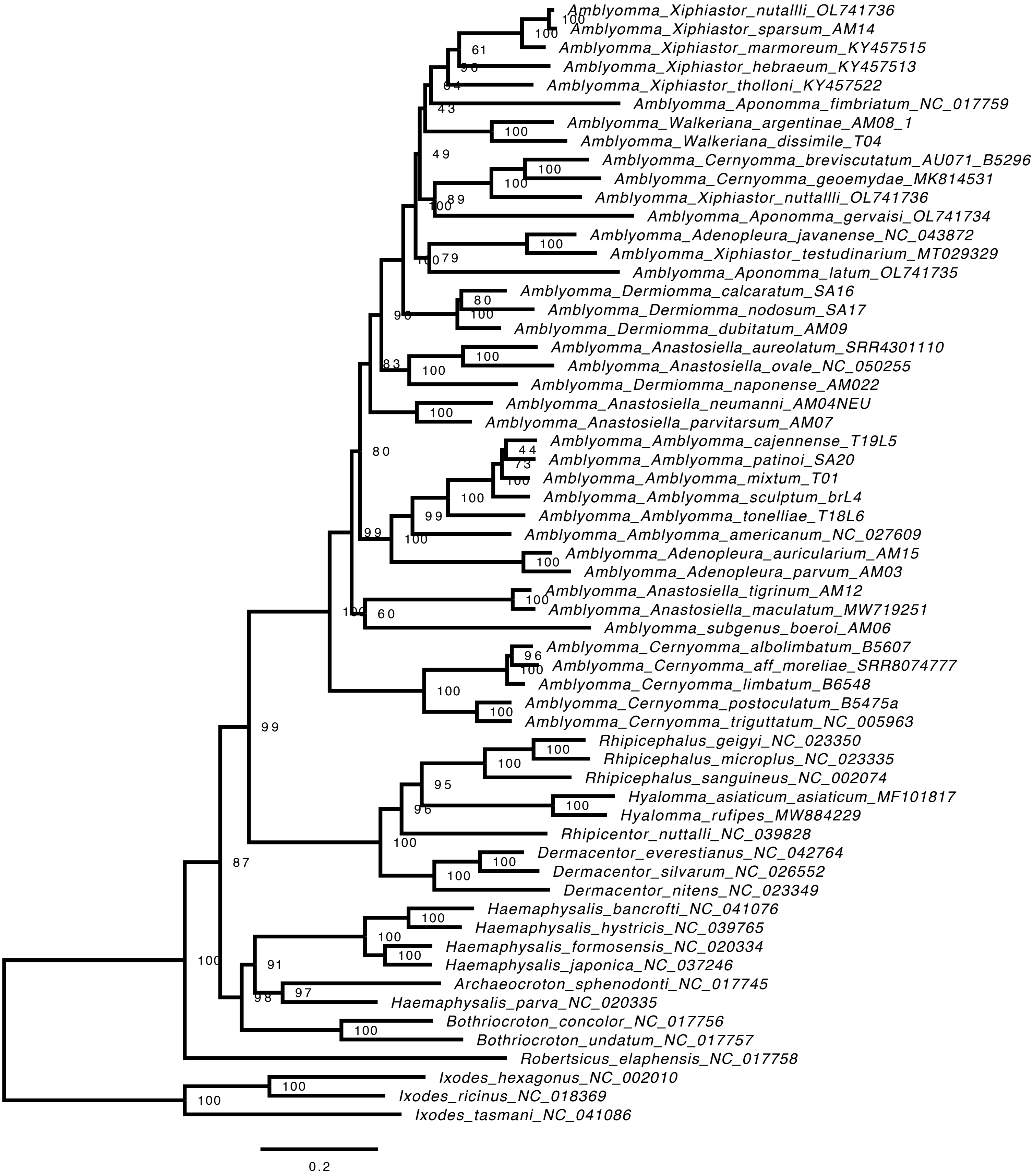
Amblyomma_Xiphiastor_nutallli_OL741736
Amblyomma_Xiphiastor_sparsum_AM14
Amblyomma_Xiphiastor_marmoreum_KY457515
Amblyomma_Xiphiastor_hebraeum_KY457513
Amblyomma_Xiphiastor_tholloni_KY457522
Amblyomma_Aponomma_fimbriatum_NC_017759
Amblyomma_Walkeriana_argentinae_AM08_1
Amblyomma_Walkeriana_dissimile_T04
Amblyomma_Cernyomma_breviscutatum_AU071_B5296
Amblyomma_Cernyomma_geoemydae_MK814531
Amblyomma_Xiphiastor_nuttallli_OL741736
Amblyomma_Aponomma_gervaisi_OL741734
Amblyomma_Adenopleura_javanense_NC_043872
Amblyomma_Xiphiastor_testudinarium_MT029329
Amblyomma_Aponomma_latum_OL741735
Amblyomma_Dermiomma_calcaratum_SA16
Amblyomma_Dermiomma_nodosum_SA17
Amblyomma_Dermiomma_dubitatum_AM09
Amblyomma_Anastosiella_aureolatum_SRR4301110
Amblyomma_Anastosiella_ovale_NC_050255
Amblyomma_Dermiomma_naponense_AM022
Amblyomma_Anastosiella_neumanni_AM04NEU
Amblyomma_Anastosiella_parvitarsum_AM07
Amblyomma_Amblyomma_cajennense_T19L5
Amblyomma_Amblyomma_patinoi_SA20
Amblyomma_Amblyomma_mixtum_T01
Amblyomma_Amblyomma_sculptum_brL4
Amblyomma_Amblyomma_tonelliae_T18L6
Amblyomma_Amblyomma_americanum_NC_027609
Amblyomma_Adenopleura_auricularium_AM15
Amblyomma_Adenopleura_parvum_AM03
Amblyomma_Anastosiella_tigrinum_AM12
Amblyomma_Anastosiella_maculatum_MW719251
Amblyomma_subgenus_boeroi_AM06
Amblyomma_Cernyomma_albolimbatum_B5607
Amblyomma_Cernyomma_aff_moreliae_SRR8074777
Amblyomma_Cernyomma_limbatum_B6548
Amblyomma_Cernyomma_postoculatum_B5475a
Amblyomma_Cernyomma_triguttatum_NC_005963
Rhipicephalus_geigyi_NC_023350
Rhipicephalus_microplus_NC_023335
Rhipicephalus_sanguineus_NC_002074
Hyalomma_asiaticum_asiaticum_MF101817
Hyalomma_rufipes_MW884229
Rhipicentor_nuttalli_NC_039828
Dermacentor_everestianus_NC_042764
Dermacentor_silvarum_NC_026552
Dermacentor_nitens_NC_023349
Haemaphysalis_bancrofti_NC_041076
Haemaphysalis_hystricis_NC_039765
Haemaphysalis_formosensis_NC_020334
Haemaphysalis_japonica_NC_037246
Archaeocroton_sphenodonti_NC_017745
Haemaphysalis_parva_NC_020335
Bothriocroton_concolor_NC_017756
Bothriocroton_undatum_NC_017757
Robertsicus_elaphensis_NC_017758
Ixodes_hexagonus_NC_002010
Ixodes_ricinus_NC_018369
Ixodes_tasmani_NC_041086
0.2

Figure S1*k*.

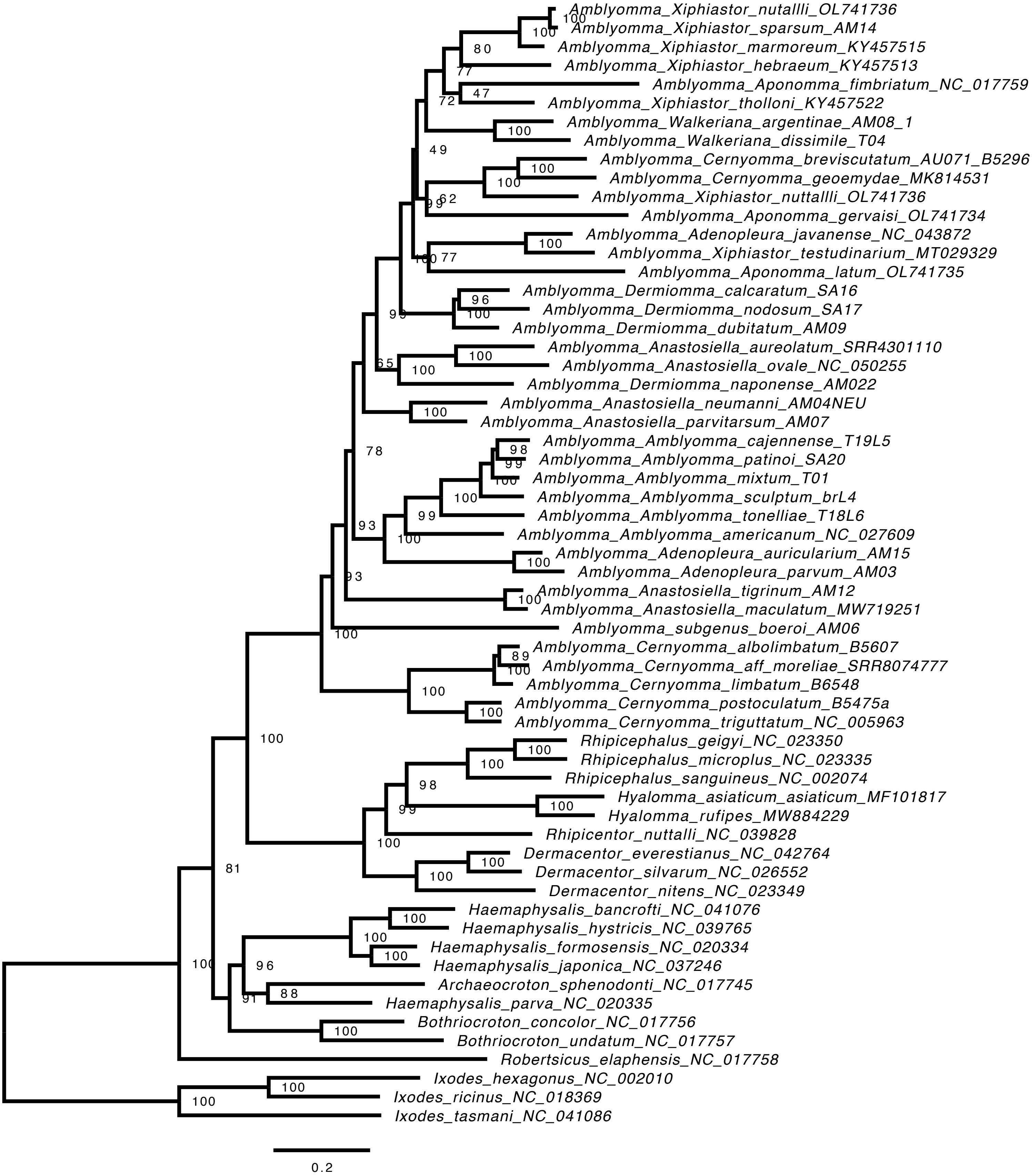
Amblyomma_Xiphiastor_nutallli_OL741736
Amblyomma_Xiphiastor_sparsum_AM14
Amblyomma_Xiphiastor_marmoreum_KY457515
Amblyomma_Xiphiastor_hebraeum_KY457513
Amblyomma_Aponomma_fimbriatum_NC_017759
Amblyomma_Xiphiastor_tholloni_KY457522
Amblyomma_Walkeriana_argentinae_AM08_1
Amblyomma_Walkeriana_dissimile_T04
Amblyomma_Cernyomma_breviscutatum_AU071_B5296
Amblyomma_Cernyomma_geoemydae_MK814531
Amblyomma_Xiphiastor_nuttallli_OL741736
Amblyomma_Aponomma_gervaisi_OL741734
Amblyomma_Adenopleura_javanense_NC_043872
Amblyomma_Xiphiastor_testudinarium_MT029329
Amblyomma_Aponomma_latum_OL741735
Amblyomma_Dermiomma_calcaratum_SA16
Amblyomma_Dermiomma_nodosum_SA17
Amblyomma_Dermiomma_dubitatum_AM09
Amblyomma_Anastosiella_aureolatum_SRR4301110
Amblyomma_Anastosiella_ovale_NC_050255
Amblyomma_Dermiomma_naponense_AM022
Amblyomma_Anastosiella_neumanni_AM04NEU
Amblyomma_Anastosiella_parvitarsum_AM07
Amblyomma_Amblyomma_cajennense_T19L5
Amblyomma_Amblyomma_patinoi_SA20
Amblyomma_Amblyomma_mixtum_T01
Amblyomma_Amblyomma_sculptum_brL4
Amblyomma_Amblyomma_tonelliae_T18L6
Amblyomma_Amblyomma_americanum_NC_027609
Amblyomma_Adenopleura_auricularium_AM15
Amblyomma_Adenopleura_parvum_AM03
Amblyomma_Anastosiella_tigrinum_AM12
Amblyomma_Anastosiella_maculatum_MW719251
Amblyomma_subgenus_boeroi_AM06
Amblyomma_Cernyomma_albolimbatum_B5607
Amblyomma_Cernyomma_aff_moreliae_SRR8074777
Amblyomma_Cernyomma_limbatum_B6548
Amblyomma_Cernyomma_postoculatum_B5475a
Amblyomma_Cernyomma_triguttatum_NC_005963
Rhipicephalus_geigyi_NC_023350
Rhipicephalus_microplus_NC_023335
Rhipicephalus_sanguineus_NC_002074
Hyalomma_asiaticum_asiaticum_MF101817
Hyalomma_rufipes_MW884229
Rhipicentor_nuttalli_NC_039828
Dermacentor_everestianus_NC_042764
Dermacentor_silvarum_NC_026552
Dermacentor_nitens_NC_023349
Haemaphysalis_bancrofti_NC_041076
Haemaphysalis_hystricis_NC_039765
Haemaphysalis_formosensis_NC_020334
Haemaphysalis_japonica_NC_037246
Archaeocroton_sphenodonti_NC_017745
Haemaphysalis_parva_NC_020335
Bothriocroton_concolor_NC_017756
Bothriocroton_undatum_NC_017757
Robertsicus_elaphensis_NC_017758
Ixodes_hexagonus_NC_002010
Ixodes_ricinus_NC_018369
Ixodes_tasmani_NC_041086
100
100
80
77
72
47
100
49
100
100
99
62
100
100
77
96
100
93
65
100
100
100
78
98
99
100
100
99
93
100
100
93
100
100
89
100
100
100
100
100
100
98
99
100
100
100
100
81
100
100
100
96
91
88
100
100
100
0.2
