# Supplementary figures and images for: New insights into the molecular phylogeny, biogeographical history, and diversification of Amblyomma ticks (Acari: Ixodidae) based on mitogenomes and nuclear sequences
Source: Parasit Vectors. 2024 Mar 18;17:139. doi: 10.1186/s13071-024-06131-w (PMC10946108; doi:10.1186/s13071-024-06131-w)

Figure S2.

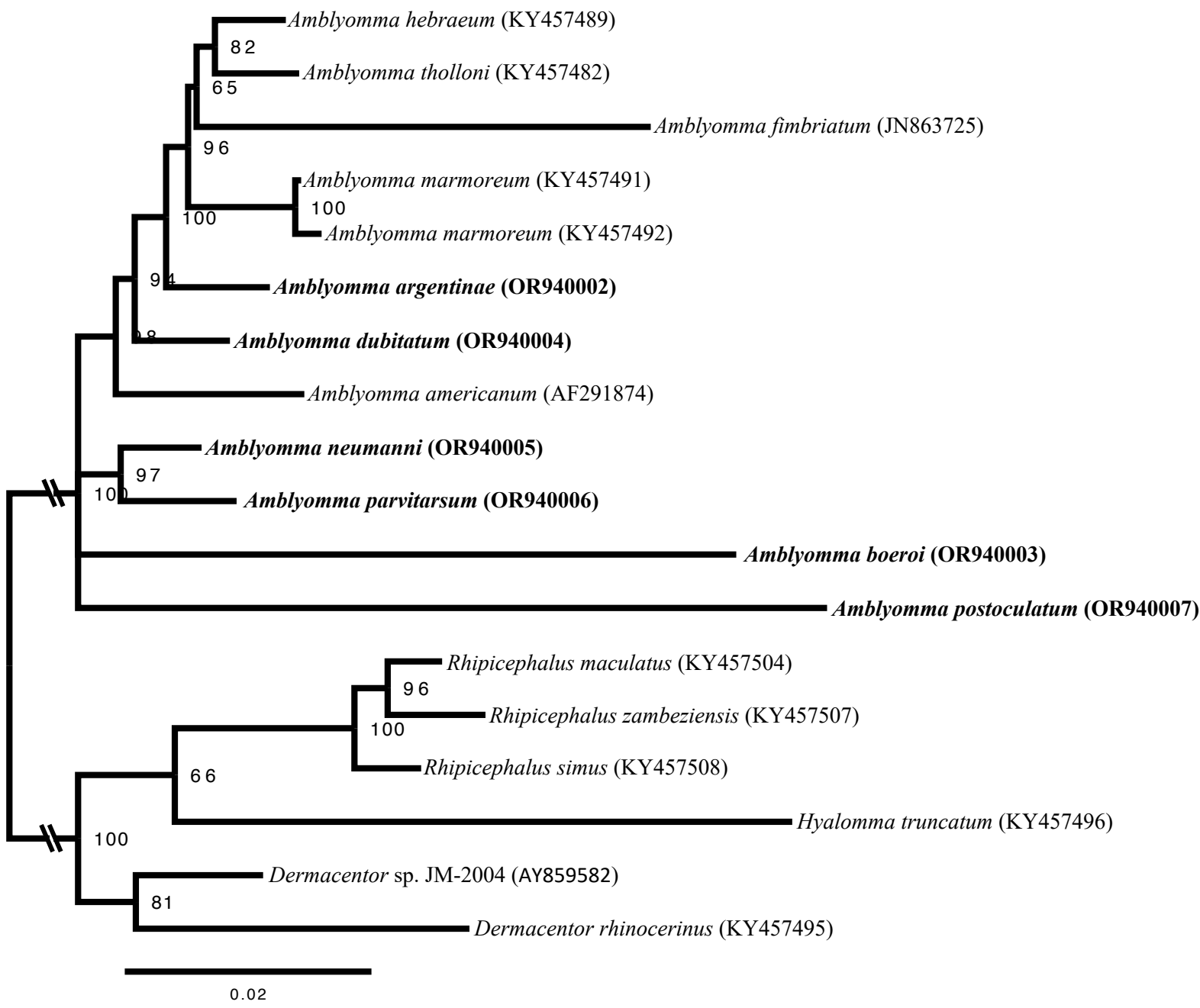

Supplement: Supplementary file 6 — Additional file 6: Figure S2. Phylogenetic tree based on nuclear ribosomal cluster. Maximum-Likelihood tree was reconstructed using the best fit evolutionary model for the concatenated matrix. Numbers at nodes are statistical support values for bootstrap proportions. The scale bar is in expected substitutions/site. The specimens sequenced in this study are highlighted in bold font with their respective GenBank access number in parentheses. [file 13071_2024_6131_MOESM6_ESM.pdf]
