# Supplementary material for: New insights into the molecular phylogeny, biogeographical history, and diversification of Amblyomma ticks (Acari: Ixodidae) based on mitogenomes and nuclear sequences
Source: Parasit Vectors. 2024 Mar 18;17:139. doi: 10.1186/s13071-024-06131-w (PMC10946108; doi:10.1186/s13071-024-06131-w)
Supplement: Supplementary file 7 — Additional file 7: Figure S3. a Biogeographic hypothesis using Biogeographic hypothesis using BioGeoBEARS DEC on garrapatas M0_unconstrained ancstates: global optim, 5 areas max. d = 0.0024; e = 0; j = 0; LnL = − 71.43. b Biogeographic hypothesis using BioGeoBEARS DEC+J on garrapatas M0_unconstrained ancstates: global optim, 5 areas max. d = 0.0018; e = 0; j = 0.0116; LnL = − 66.07. c Biogeographic hypothesis using BioGeoBEARS DIVALIKE on Psychotria M0_unconstrained ancstates: global optim, 5 areas max. d = 0.0028; e = 0; j = 0; LnL = − 69.79. d Biogeographic hypothesis using BioGeoBEARS DIVALIKE+J on Psychotria M0_unconstrained ancstates: global optim, 5 areas max. d = 0.0019; e = 0; j = 0.0104; LnL = − 66.16. Note: In all scenarios (a–d) five stages were assessed, each of them indicated with its respective abbreviation as follows: Nearctic, NE; Neotropic, NO; Australia, AU; Afrotropic, AF; Indomalayan, IN. [file 13071_2024_6131_MOESM7_ESM.pdf]

Figure S3*a*.

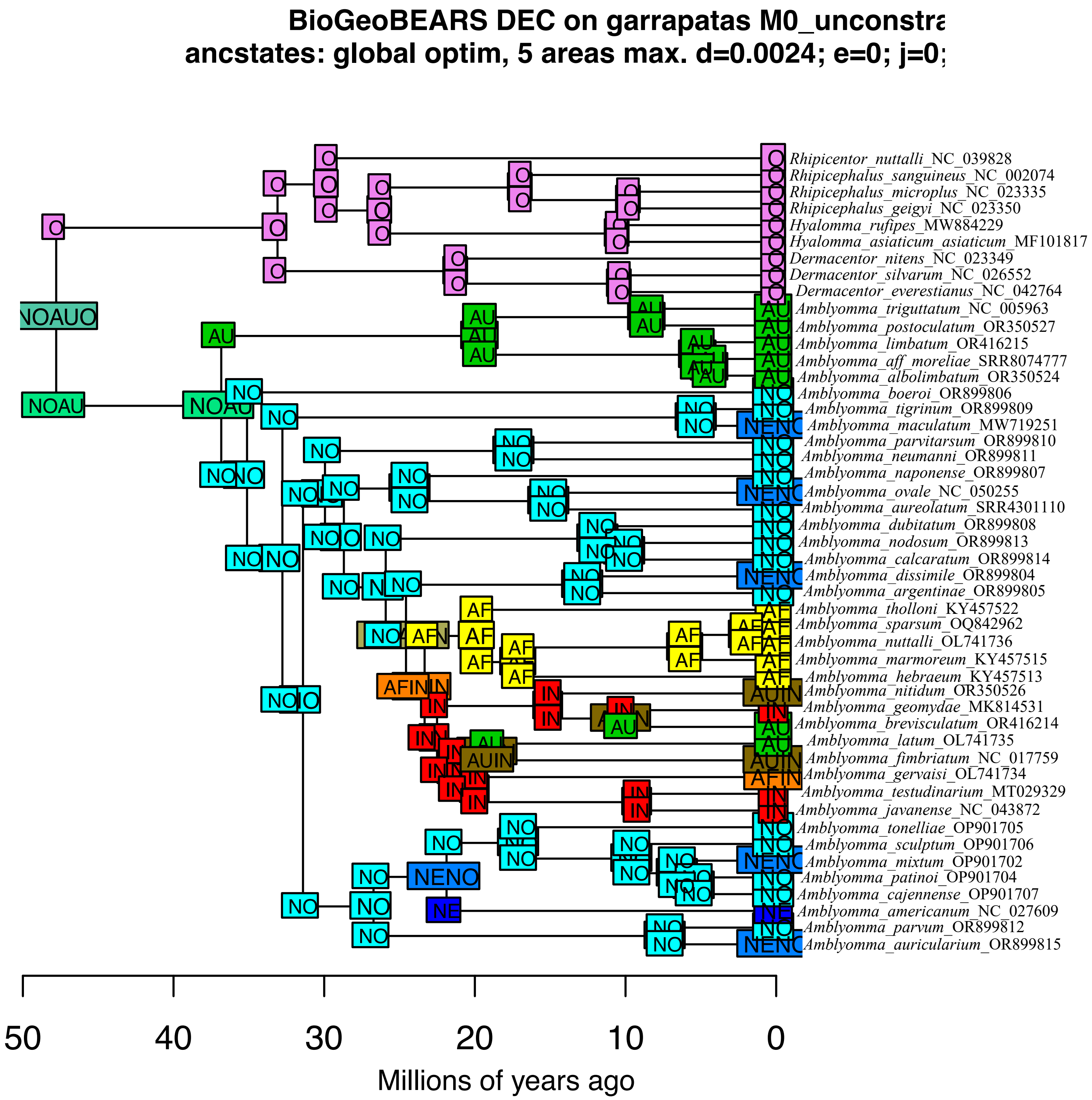
BioGeoBEARS DEC on garrapatas M0_unconstra
ancstates: global optim, 5 areas max. d=0.0024; e=0; j=0;
Rhipicentor_nuttalli_NC_039828
Rhipicephalus_sanguineus_NC_002074
Rhipicephalus_microplus_NC_023335
Rhipicephalus_geigyi_NC_023350
Hyalomma_rufipes_MW884229
Hyalomma_asiaticum_asiaticum_MF101817
Dermacentor_nitens_NC_023349
Dermacentor_silvarum_NC_026552
Dermacentor_everestianus_NC_042764
Amblyomma_triguttatum_NC_005963
Amblyomma_postoculatum_OR350527
Amblyomma_limbatum_OR416215
Amblyomma_aff_moreliae_SRR8074777
Amblyomma_albolimbatum_OR350524
Amblyomma_boeroi_OR899806
Amblyomma_tigrinum_OR899809
Amblyomma_maculatum_MW719251
Amblyomma_parvitarsum_OR899810
Amblyomma_neumanni_OR899811
Amblyomma_naponense_OR899807
Amblyomma_ovale_NC_050255
Amblyomma_aureolatum_SRR4301110
Amblyomma_dubitatum_OR899808
Amblyomma_nodosum_OR899813
Amblyomma_calcaratum_OR899814
Amblyomma_dissimile_OR899804
Amblyomma_argentinae_OR899805
Amblyomma_tholloni_KY457522
Amblyomma_sparsum_OQ842962
Amblyomma_nuttalli_OL741736
Amblyomma_marmoreum_KY457515
Amblyomma_hebraeum_KY457513
Amblyomma_nitidum_OR350526
Amblyomma_geomydae_MK814531
Amblyomma_breviscutatum_OR416214
Amblyomma_latum_OL741735
Amblyomma_fimbriatum_NC_017759
Amblyomma_gervaisi_OL741734
Amblyomma_testudinarium_MT029329
Amblyomma_javanense_NC_043872
Amblyomma_tonelliae_OP901705
Amblyomma_sculptum_OP901706
Amblyomma_mixtum_OP901702
Amblyomma_patinoi_OP901704
Amblyomma_cajennense_OP901707
Amblyomma_americanum_NC_027609
Amblyomma_parvum_OR899812
Amblyomma_auricularium_OR899815
50
40
30
20
10
0
Millions of years ago

Figure S3*b*.

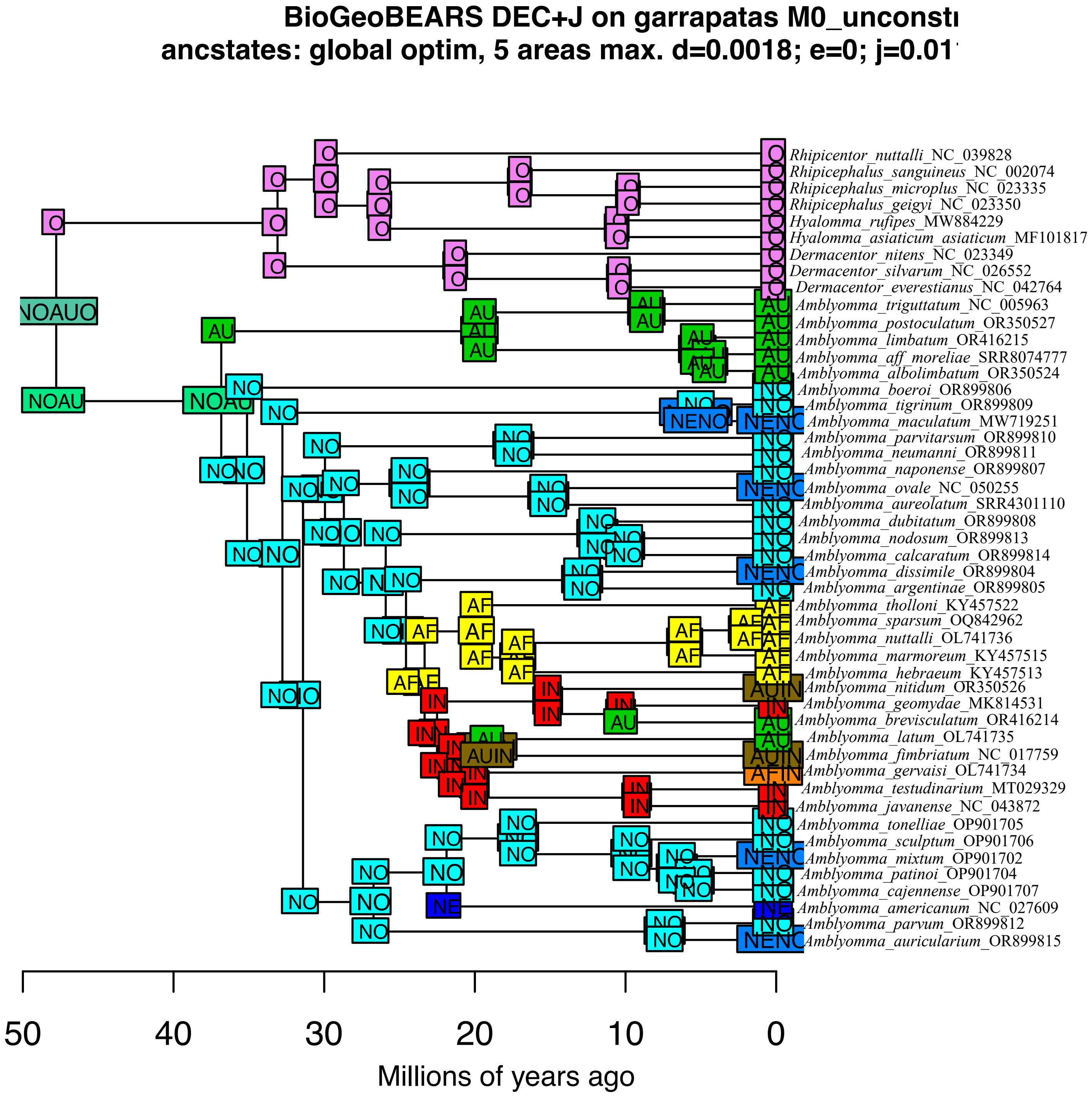
BioGeoBEARS DEC+J on garrapatas M0_unconst
ancstates: global optim, 5 areas max. d=0.0018; e=0; j=0.01
Rhipicentor_nuttalli_NC_039828
Rhipicephalus_sanguineus_NC_002074
Rhipicephalus_microplus_NC_023335
Rhipicephalus_geigyi_NC_023350
Hyalomma_rufipes_MW884229
Hyalomma_asiaticum_asiaticum_MF101817
Dermacentor_nitens_NC_023349
Dermacentor_silvarum_NC_026552
Dermacentor_everestianus_NC_042764
Amblyomma_triguttatum_NC_005963
Amblyomma_postoculatum_OR350527
Amblyomma_limbatum_OR416215
Amblyomma_aff_moreliae_SRR8074777
Amblyomma_albolimbatum_OR350524
Amblyomma_boeroi_OR899806
Amblyomma_tigrinum_OR899809
Amblyomma_maculatum_MW719251
Amblyomma_parvitarsum_OR899810
Amblyomma_neumanni_OR899811
Amblyomma_naponense_OR899807
Amblyomma_ovale_NC_050255
Amblyomma_aureolatum_SRR4301110
Amblyomma_dubitatum_OR899808
Amblyomma_nodosum_OR899813
Amblyomma_calcaratum_OR899814
Amblyomma_dissimile_OR899804
Amblyomma_argentinae_OR899805
Amblyomma_tholloni_KY457522
Amblyomma_sparsum_OQ842962
Amblyomma_nuttalli_OL741736
Amblyomma_marmoreum_KY457515
Amblyomma_hebraeum_KY457513
Amblyomma_nitidum_OR350526
Amblyomma_geomydae_MK814531
Amblyomma_breviscutatum_OR416214
Amblyomma_latum_OL741735
Amblyomma_fimbriatum_NC_017759
Amblyomma_gervaisi_OL741734
Amblyomma_testudinarium_MT029329
Amblyomma_javanense_NC_043872
Amblyomma_tonelliae_OP901705
Amblyomma_sculptum_OP901706
Amblyomma_mixtum_OP901702
Amblyomma_patinoi_OP901704
Amblyomma_cajennense_OP901707
Amblyomma_americanum_NC_027609
Amblyomma_parvum_OR899812
Amblyomma_auricularium_OR899815
50
40
30
20
10
0
Millions of years ago

Figure S3*c*.

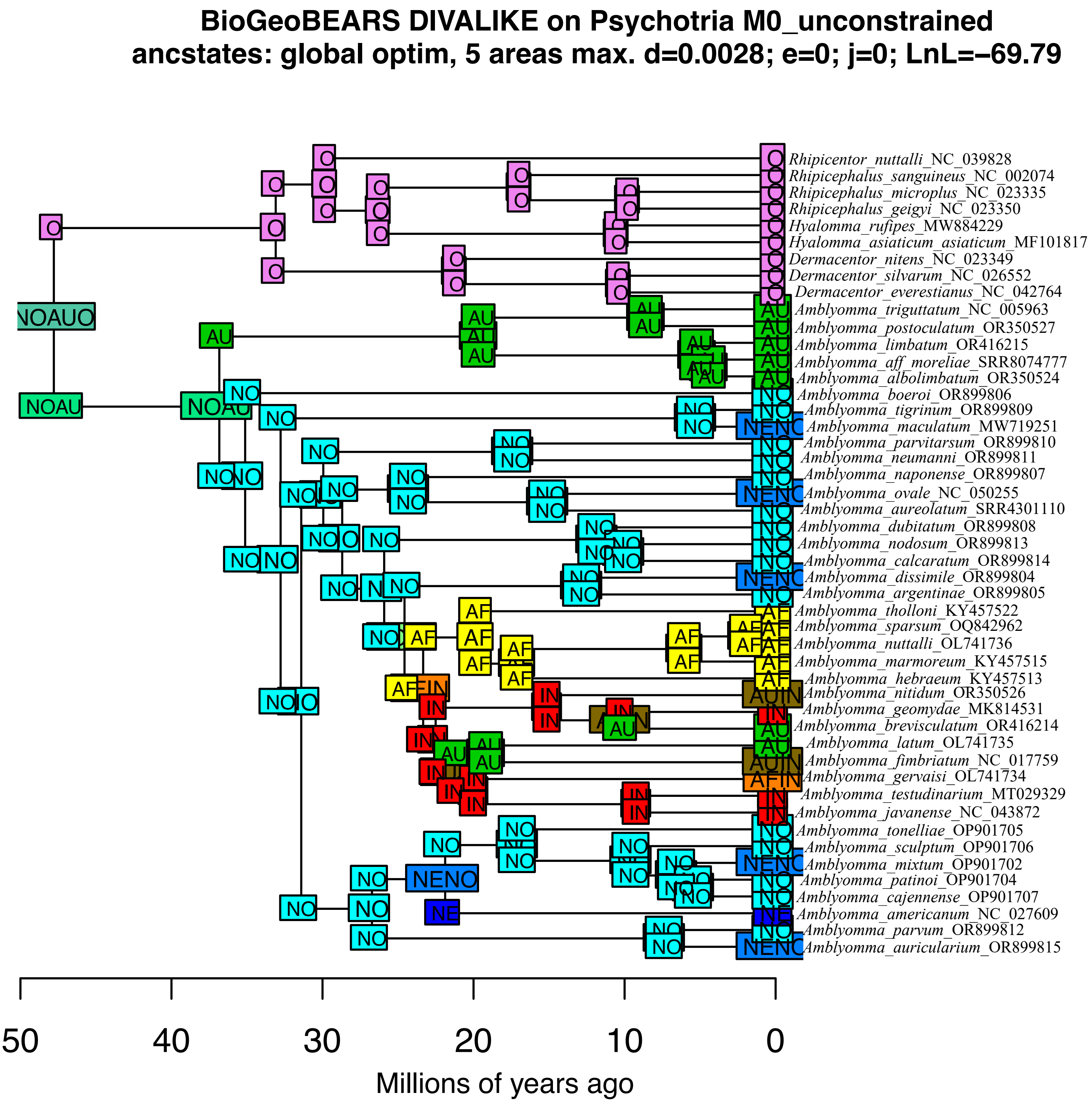

BioGeoBEARS DIVALIKE on Psychotria M0_unconstrained
ancstates: global optim, 5 areas max. d=0.0028; e=0; j=0; LnL=-69.79
Rhipicentor_nuttalli_NC_039828
Rhipicephalus_sanguineus_NC_002074
Rhipicephalus_microplus_NC_023335
Rhipicephalus_geigyi_NC_023350
Hyalomma_rufipes_MW884229
Hyalomma_asiaticum_asiaticum_MF101817
Dermacentor_nitens_NC_023349
Dermacentor_silvarum_NC_026552
Dermacentor_everestianus_NC_042764
Amblyomma_triguttatum_NC_005963
Amblyomma_postoculatum_OR350527
Amblyomma_limbatum_OR416215
Amblyomma_aff_moreliae_SRR8074777
Amblyomma_albolimbatum_OR350524
Amblyomma_boeroi_OR899806
Amblyomma_tigrinum_OR899809
Amblyomma_maculatum_MW719251
Amblyomma_parvitarsum_OR899810
Amblyomma_neumanni_OR899811
Amblyomma_naponense_OR899807
Amblyomma_ovale_NC_050255
Amblyomma_aureolatum_SRR4301110
Amblyomma_dubitatum_OR899808
Amblyomma_nodosum_OR899813
Amblyomma_calcaratum_OR899814
Amblyomma_dissimile_OR899804
Amblyomma_argentinae_OR899805
Amblyomma_tholloni_KY457522
Amblyomma_sparsum_OQ842962
Amblyomma_nuttalli_OL741736
Amblyomma_marmoreum_KY457515
Amblyomma_hebraeum_KY457513
Amblyomma_nitidum_OR350526
Amblyomma_geomydae_MK814531
Amblyomma_breviscutatum_OR416214
Amblyomma_latum_OL741735
Amblyomma_fimbriatum_NC_017759
Amblyomma_gervaisi_OL741734
Amblyomma_testudinarium_MT029329
Amblyomma_javanense_NC_043872
Amblyomma_tonelliae_OP901705
Amblyomma_sculptum_OP901706
Amblyomma_mixtum_OP901702
Amblyomma_patinoi_OP901704
Amblyomma_cajennense_OP901707
Amblyomma_americanum_NC_027609
Amblyomma_parvum_OR899812
Amblyomma_auricularium_OR899815
50
40
30
20
10
0
Millions of years ago

Figure S3*d*.

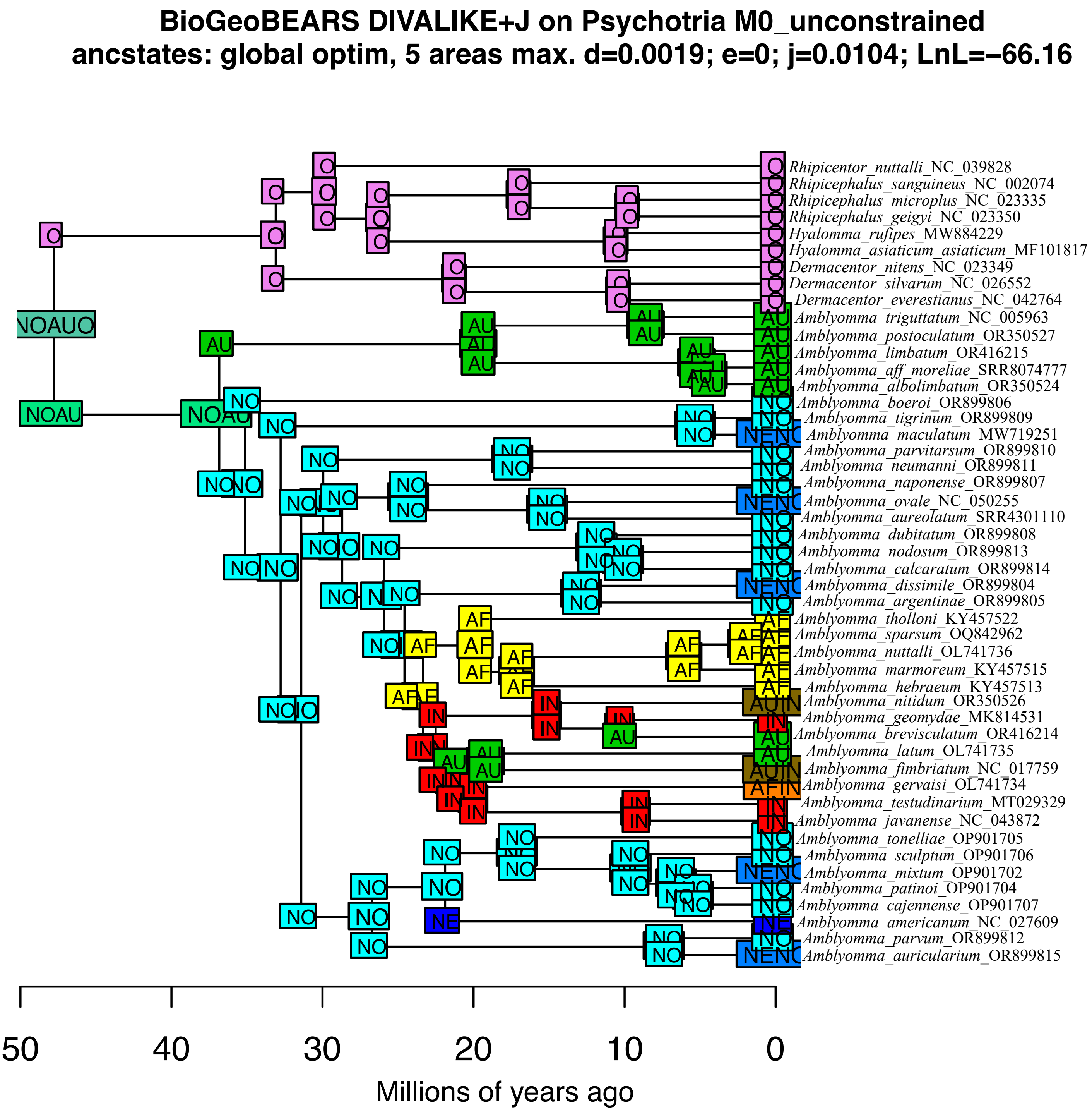

BioGeoBEARS DIVALIKE+J on Psychotria M0_unconstrained
ancstates: global optim, 5 areas max. d=0.0019; e=0; j=0.0104; LnL=-66.16
Rhipicentor_nuttalli_NC_039828
Rhipicephalus_sanguineus_NC_002074
Rhipicephalus_microplus_NC_023335
Rhipicephalus_geigyi_NC_023350
Hyalomma_rufipes_MW884229
Hyalomma_asiaticum_asiaticum_MF101817
Dermacentor_nitens_NC_023349
Dermacentor_silvarum_NC_026552
Dermacentor_everestianus_NC_042764
Amblyomma_triguttatum_NC_005963
Amblyomma_postoculatum_OR350527
Amblyomma_limbatum_OR416215
Amblyomma_aff_moreliae_SRR8074777
Amblyomma_albolimbatum_OR350524
Amblyomma_boeroi_OR899806
Amblyomma_tigrinum_OR899809
Amblyomma_maculatum_MW719251
Amblyomma_parvitarsum_OR899810
Amblyomma_neumanni_OR899811
Amblyomma_naponense_OR899807
Amblyomma_ovale_NC_050255
Amblyomma_aureolatum_SRR4301110
Amblyomma_dubitatum_OR899808
Amblyomma_nodosum_OR899813
Amblyomma_calcaratum_OR899814
Amblyomma_dissimile_OR899804
Amblyomma_argentinae_OR899805
Amblyomma_tholloni_KY457522
Amblyomma_sparsum_OQ842962
Amblyomma_nuttalli_OL741736
Amblyomma_marmoreum_KY457515
Amblyomma_hebraeum_KY457513
Amblyomma_nitidum_OR350526
Amblyomma_geomydae_MK814531
Amblyomma_breviscutatum_OR416214
Amblyomma_latum_OL741735
Amblyomma_fimbriatum_NC_017759
Amblyomma_gervaisi_OL741734
Amblyomma_testudinarium_MT029329
Amblyomma_javanense_NC_043872
Amblyomma_tonelliae_OP901705
Amblyomma_sculptum_OP901706
Amblyomma_mixtum_OP901702
Amblyomma_patinoi_OP901704
Amblyomma_cajennense_OP901707
Amblyomma_americanum_NC_027609
Amblyomma_parvum_OR899812
Amblyomma_auricularium_OR899815
50
40
30
20
10
0
Millions of years ago
